# Comprehensive overview of COVID-19-related respiratory failure: focus on cellular interactions

**DOI:** 10.1186/s11658-022-00363-3

**Published:** 2022-07-30

**Authors:** Fahimeh Zamani Rarani, Mohammad Zamani Rarani, Michael R. Hamblin, Bahman Rashidi, Seyed Mohammad Reza Hashemian, Hamed Mirzaei

**Affiliations:** 1 Department of Anatomical Sciences, School of Medicine, Isfahan University of Medical Sciences, Isfahan, Iran; 2 Laser Research Centre, Faculty of Health Science, University of Johannesburg, Doornfontein, 2028 South Africa; 3 Chronic Respiratory Diseases Research Center (CRDRC), National Research Institute of Tuberculosis and Lung Diseases (NRITLD), Shahid Beheshti University of Medical Sciences, Tehran, Iran; 4 Student Research Committee, Kashan University of Medical Sciences, Kashan, Iran; 5 Research Center for Biochemistry and Nutrition in Metabolic Diseases, Institute for Basic Sciences, Kashan University of Medical Sciences, Kashan, IR Iran

**Keywords:** SARS-CoV-2, COVID-19, Airway epithelial cells, Endothelial cells, Platelets, Cytokines, Pulmonary edema

## Abstract

The pandemic outbreak of coronavirus disease 2019 (COVID-19) has created health challenges in all parts of the world. Understanding the entry mechanism of this virus into host cells is essential for effective treatment of COVID-19 disease. This virus can bind to various cell surface molecules or receptors, such as angiotensin-converting enzyme 2 (ACE2), to gain cell entry. Respiratory failure and pulmonary edema are the most important causes of mortality from COVID-19 infections. Cytokines, especially proinflammatory cytokines, are the main mediators of these complications. For normal respiratory function, a healthy air–blood barrier and sufficient blood flow to the lungs are required. In this review, we first discuss airway epithelial cells, airway stem cells, and the expression of COVID-19 receptors in the airway epithelium. Then, we discuss the suggested molecular mechanisms of endothelial dysfunction and blood vessel damage in COVID-19. Coagulopathy can be caused by platelet activation leading to clots, which restrict blood flow to the lungs and lead to respiratory failure. Finally, we present an overview of the effects of immune and non-immune cells and cytokines in COVID-19-related respiratory failure.

## Background

Coronavirus disease 2019 (COVID-19) was first identified in Wuhan, China in December 2019, and then rapidly spread to all parts of the world. The World Health Organization (WHO), on 11 March, denoted COVID-19 as a pandemic, which was caused by novel severe acute respiratory syndrome coronavirus 2 (SARS-CoV-2) [1–7].

Human coronaviruses (CoVs) were first reported in 1962. On the basis of the International Committee on Taxonomy of Viruses (ICTV), CoVs belong to Riboviria realm, Nidovirales order, Coronaviridae family, Orthocoronavirinae subfamily. CoVs are enveloped positive single-stranded RNA viruses classified into four genera, based on their protein sequence: alpha CoVs, beta CoVs, gamma CoVs, and delta CoVs. The intermediate hosts for alpha- and beta-coronaviruses are bats and rodents, whereas birds fulfill this role for gamma- and delta-coronaviruses [8–10].

Beta-coronaviruses are categorized into A, B, C, and D lineages, which cause most human coronavirus infections. Up to now, six beta-coronaviruses have been identified: HCoV-OC43, HCoV-HKU1, HCoV-229E, MERS-CoV, SARS-CoV, and SARS-CoV-2 [9, 10].

Over the past two decades, three epidemic or pandemic outbreaks of CoV infection have occurred (SARS-CoV in 2002, MERS-CoV in 2012, and SARS-CoV-2 from 2019 onward). All these viruses were capable of infecting human airways, especially the lower respiratory tract, and causing acute respiratory distress syndrome (ARDS) and multiorgan failure (MOF), with high mortality rates [1, 11, 12].

Common clinical symptoms in patients with COVID-19 include nonproductive cough, fever, myalgia, fatigue, diarrhea, hypoxemia, and dyspnea, often leading to ARDS and/or multiple organ dysfunction syndrome (MODS). Whereas some patient have none or only mild symptoms, others may be prone to more serious COVID-19 infections, and then require hospitalization and intensive care [13, 14]. Pneumonia and respiratory failure is the most prevalent pathological cause of death in COVID-19, and in patients who require mechanical ventilation, mortality can be very high [15, 16].

SARS-CoV-2 entry into human cells is mediated mainly by the viral spike (S) protein. Airway epithelial cells, ciliated cells, goblet cells, and, more recently, olfactory neurons are the most studied routes for viral entry. When the virus infects these epithelial cells, the pathogenic process commences, and in favorable conditions the virus begins to multiply rapidly, then spread to infect other target cells and organs. Angiotensin-converting enzyme 2 (ACE2) is the most common receptor for SARS-CoV-2 S protein, but recently other receptors have been identified that can interact with S protein [17–19].

During COVID-19 infections, the air–blood barrier can be disturbed by apoptosis or necrosis of epithelial cells and endothelial cells, as well as damage to the basement membrane. This airway damage can lead to respiratory failure and death. On the other hand, it has been suggested that COVID-19 is largely an endothelial disease. It is known that there are different receptors and other mediators expressed in endothelial cells, which can help SARS-CoV-2 to gain entry [20–24]. When the SARS-CoV-2 virus infects endothelial cells, it affects their secreted mediators, tight cell junctions, and overall survival [25].

It has been reported that coagulopathy is commonly observed in patients with severe COVID-19, and that endothelial cells as well as platelets play a key role in this process. Similar to endothelial cells, platelets express receptors and secretory granules, which can be affected by the pathogen [26–29].

SARS-CoV-2 can affect target cells either directly by binding to surface receptors, or indirectly by cytokines secreted from other cells. Cytokines are signaling molecules secreted by a wide range of cell types. They are critically involved in many biological processes. Cytokines have either proinflammatory or anti-inflammatory effects. These molecules can produce a “cytokine storm” or overproduction of proinflammatory cytokines leading to further damage, especially involving feedback loops. The cytokine storm has been widely reviewed [30–32].

The SARS-CoV-2 pathogenesis process involves a variety of cells, which can interact with each other and with cytokines, leading to the destruction of the air–blood barrier and causing coagulopathy. This can eventually lead to pulmonary edema, ARDS and/or MODS, and finally death. Our goal in this article is to provide a comprehensive overview of the cells and processes involved in COVID-19-related respiratory failure. Firstly, we discuss the pulmonary epithelium and SARS-CoV-2 entry mechanism, followed by the influence of COVID-19 on endothelial cells and platelets, and finally we suggest how all these factors interact together, to understand the pathophysiology of pulmonary complications induced by SARS-CoV-2 infection.

### Pulmonary epithelium

**Fig. 1:**
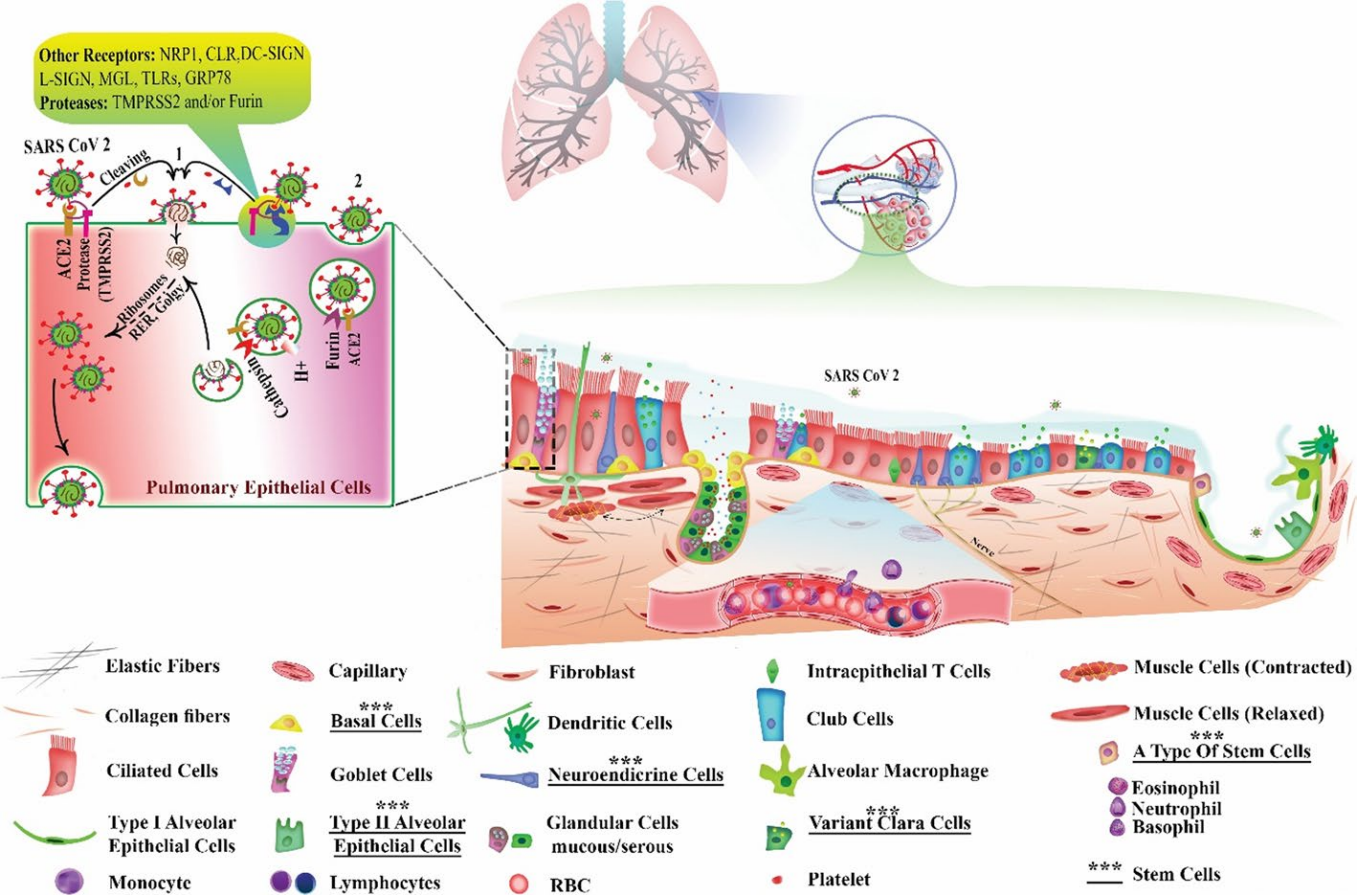
Pulmonary epithelium and cell penetration pathways of SARS-CoV-2. **A** One of the important ports of virus entry is respiratory epithelial cells. Upper airways are lined with pseudostratified epithelium. In distal airways, height of the epithelium decreases and eventually becomes squamous in the alveoli. It consists mainly of ciliated cells as well as goblet cells, Clara/club cells, basal cells, and neuroendocrine cells. Ciliated cells have hair-like projections, which help move up mucus that rests on them. Goblet cells produce and secrete mucin. Club cells secret specific proteins and surfactant protein (SP)-A, SP-B, and SP-D. Alveolar type 1 and 2 cells are involved in gas exchange and the generation of SPs, respectively. Stem cells in this epithelium include basal cells, “variant” club cells, neuroendocrine cells, and cell population in bronchoalveolar duct junctions. **B** SARS‐CoV‐2 entry into the host cells occurs via direct membrane fusion (1) and endocytosis (2). In both pathways, spike (S) protein must bind to host cell receptors such as ACE2, NRP1, CLR, MGL, L-SIGN, DC-SIGN, TLRs, and GRP78. In endocytosis-mediated entry, following binding to cell receptor, virus entry into the host cell occurs and the S protein is activated in endosomes by furin cleavage. Fusion occurs by cathepsin-L action, and virus genetic material is released into cytosol, entering the virus through direct fusion mediated by proteases such as TMPRSS2 and/or furin. S protein interacts with a host cell receptor and becomes activated. Eventually, the membranes are merged and the virus releases its genetic material (RNA) via the formed pore into the cytosol. SARS-CoV-2 RNA is replicated and transcribed by host organelles such as ribosomes, Golgi apparatus, rough endoplasmic reticulum (rER), etc. Finally, the virus spreads to other cells and tissues. SARS-CoV-2, severe acute respiratory syndrome coronavirus 2; NRP1, neuropilin-1; ACE2, angiotensin-converting enzyme 2; MGL, macrophage galactose-type lectin; CLR, C-lectin type receptors; L-SIGN, homolog dendritic cell-specific intercellular adhesion molecule-3-grabbing non-integrin related; DC-SIGN, dendritic cell-specific intracellular adhesion molecule-3-grabbing non-integrin; TLRs, Toll-like receptors; GRP78, non-immune receptor glucose-regulated protein 78; TMPRSS2, transmembrane protease, serine 2

The airway epithelium is vital for its varied functions, such as warming and humidifying the inhaled air, clearing and defending the airways from inspired pathogens, and forming the epithelial half of the air–blood barrier. The most important point of SARS-COVID-2 entry is the respiratory tract, and epithelial cells are the most important targets of this virus. Therefore, it is necessary to review the various cell types in the respiratory epithelium (Fig. 1).

A pseudostratified epithelium covers the upper airways, consisting of basal cells, ciliated cells as the main cell type, goblet cells, Clara or club cells, and a limited number of neuroendocrine cells [33, 34]. Columnar ciliated cells have 200–300 individual cilia and are the main cell type in this epithelium. Their hair-like projections move backwards and forwards at a rapid frequency (8–20 Hz) and help move mucus upwards through the tract. Goblet cells are also columnar, like ciliated cells. They produce and secrete mucin, a glycoprotein, into the airway for trapping inhaled particles and pathogens, also for and moisturizing the epithelium [33, 35]. Clara or club cells are cube-shaped cells that secret proteins (e.g., CCSP) and surfactant proteins (SP)-A, SP-B, and SP-D. These proteins are involved in the composition of lung fluid [33, 36].

In distal airways and bronchioles, the Clara cells become more abundant and the lung epithelium gradually becomes more columnar in nature. The height of the epithelium decreases and eventually becomes squamous within the alveoli. Alveolar type 1 and 2 (AT1, AT2) cells are two important cell types in the alveolar epithelium. They are involved in gas exchange and surfactant production, respectively [37, 38].

### Airway stem cells

The most important cause of respiratory failure and alveolar edema in COVID-19 is destruction of the integrity of the pulmonary epithelium. Airway stem cells have a crucial role in maintaining and repairing the epithelial integrity. The self-renewing stem cells are able to generate several different types of daughter cells [39, 40]. Owing to the difficulties of direct studies in human lung, there is insufficient knowledge about the human lung epithelial stem cell niche. Knowledge on the repair and maintenance of lung function has been largely based on animal studies. In animals, airway stem cells are found in three niches within the tract: upper airway submucosal glands, small airway branching points, and the bronchoalveolar duct junction (BADJ). The cells in these niches are capable of generating various cell types [40].

These include: basal cells, “variant” Clara cells, neuroendocrine cells, and the cell population in the BADJ. BADJ stem cells can differentiate into AT1, Clara, and AT2 cells [41–43]. It has been found that AT2 cells can replace lost AT1 cells in the alveolar epithelium (Fig. 1) [44]. Further studies should be performed on the function of these cells in humans, and whether they can help to treat respiratory failure in patients with COVID-19 and control its subsequent complications.

### Cell entry mechanisms of SARS-CoV-2

The coronavirus virion is made up of nucleocapsid (N), membrane (M), envelope (E), and spike (S) proteins, which are structural proteins. The S protein mediates viral attachment, membrane fusion, and entry, thus determining tissue and cell tropism as well as host range in the Coronaviridae family [45]. Therefore, coronavirus infection of target cells depends on interactions of S proteins with cellular receptors [46]; for example, MHV uses murine biliary glycoproteins in the immunoglobulin superfamily; HCoV-229E and TGEV use human and porcine aminopeptidase N (APN), respectively; FIPV and FeCoV use feline APN, which can also be utilized by HCoV-229E and TGEV; and BCoV and HCoV-OC43 use *N*-acetyl-9-*O*-acetyl neuraminic acid moieties. Expression of the cloned receptor glycoproteins in cells of a foreign species can render them susceptible to infection with coronavirus virions [45]. Thus, coronavirus–receptor interactions are an important determinant of the species specificity of coronavirus infection.

SARS‐CoV‐2 enters the host cells via endocytosis of a receptor–virus complex, or by direct membrane fusion (Fig. 1). In both pathways, the SARS-CoV-2 S protein, a trimeric class I transmembrane glycoprotein, must bind to cell receptors like ACE2 in the host. Respiratory epithelial and endothelial cells, monocytes, and alveolar macrophages show broad expression of ACE2 [47, 48].

In addition to ACE2, several molecules have been suggested to serve as alternative receptors for SARS-CoV and SARS-CoV-2. These include C-type lectins, DC-SIGN and L-SIGN. Lectins are involved in the recognition of a broad range of pathogens and mediate intercellular adhesion. Although lectins and phosphatidylserine receptors enhance viral entry, they are nonspecific and do not support efficient infection by SARS-CoV or SARS-CoV-2 in the absence of ACE2, and thus “attachment factors” would better describe these molecules. Similarly, CD147, a transmembrane glycoprotein expressed ubiquitously in epithelial and immune cells, was proposed to be an alternative receptor for SARS-CoV and SARS-CoV-2 infection [49]. Although a modest increase in viral entry was observed with higher levels of CD147, and although its upregulation was observed in obesity and diabetes [49], which are potential risk factors for severe COVID-19, the role of CD147 in SARS-CoV-2 infection has been disputed on the basis of the inability of CD147 to bind the S protein [50]. Two groups identified neuropilin 1 (NRP1) as a host factor for SARS-CoV-2 [51, 52]. Although NRP1 is expressed in olfactory and respiratory epithelial cells, its expression is low in ciliated cells, the primary target cells for SARS-CoV-2 in the airway, while it is high in goblet cells, which are not susceptible to SARS-CoV-2. Nonetheless, NRP1 was shown to enhance TMPRSS2-mediated entry (see the next section) of wild-type SARS-CoV-2 but not that of mutant virus that lacks the multibasic furin-cleavage site [49]. NRP1 was also shown to bind S1 through the multibasic furin-cleavage site and to promote S1 shedding and expose the S2′ site to TMPRSS2 [53]. Recently, the structure of ACE2 in complex with a neutral amino acid transporter, B0AT1, was analyzed by cryogenic electron microscopy in the presence and absence of the SARS-CoV-2 S protein [54]. ACE2 was previously shown to be essential for B0AT1 expression in the small intestine [55]. While B0AT1 is expressed in the gastrointestinal tract and kidney, it is not present in the lung. However, a B0AT1 homolog in the lung might contribute to SARS-CoV-2 infection. Additional studies are warranted to confirm the role of NRP1 and B0AT1 in SARS-CoV-2 infection [49].

Toll-like receptors (TLRs) recognize pathogen-associated molecular patterns and induce the production of type I interferons. Of these, TLR3, TLR7, TLR8, and TLR9 mount antiviral immune responses: TLR3 recognizes double-stranded RNA viruses, TLR9 recognizes unmethylated CpG in viral DNA, and, relevant to coronaviruses, TLR7 and TLR8 bind G/U-rich single-stranded viral RNA [49]. Many interferon-stimulated gene products were identified as important for SARS-CoV-2 replication, but only a few of them are involved in the entry steps: interferon-induced transmembrane proteins (IFITMs) [56, 57] and lymphocyte antigen 6 family member E (LY6E) [58, 59]. Recently, IFITM2 was shown to restrict SARS-CoV-2 entry [56]. IFITM proteins prevent viruses from traversing the endosomal membrane to access cellular cytoplasm by an unclear mechanism. Such a restriction can be bypassed if SARS-CoV were directed to enter cells exclusively at the plasma membrane [60].

LY6E is a glycophosphatidylinositol-anchored cell surface protein and was shown to inhibit or promote replication of some viruses [49]. Recently, LY6E was shown to impair infection by SARS-CoV, SAR-CoV-2, and MERS-CoV by inhibiting the S-protein-mediated membrane fusion, and mice lacking LY6E expression in immune cells were highly susceptible to mouse hepatitis virus, also a coronavirus [59]. Unlike IFITM-mediated restriction, LY6E-mediated inhibition was not overcome by TMPRSS2 expression (58). Further study is warranted to clarify the distinct roles of LY6E in regulating infection with SARS-CoV-2 and other viruses.

In endocytosis-mediated entry, after binding to a cell receptor, the virus penetrates into the host cells, and the S protein is activated in endosomes by furin cleavage. The endosomes undergo fusion with lysosomes by the action of cathepsin L proteolysis to form the endolysosome stage. Finally, after the fusion of membranes is completed, the viral RNA enters the host cell cytosol. Proteases are responsible for allowing viral entry through direct fusion, including transmembrane protease serine 2 (TMPRSS2) or furin. After the S protein interacts with the host cell receptor, S protein activation occurs. Eventually, the membranes are merged and the virus transfer its RNA through the formed pore into the cytosol [61, 62].

The RNA of SARS-CoV-2 is translated into structural proteins and nonstructural proteins (NSPs). These proteins are necessary for virus survival, multiplication, and virulence. In addition to the S protein other structural proteins, such as envelope glycoprotein (E), membrane glycoprotein (M), and nucleocapsid (N) proteins, are synthesized as accessory proteins [63].

The replication and transcription of the RNA sequence of SARS-CoV-2 occurs by a large multisubunit viral replicase–transcriptase complex. The structural proteins and genomic material are packaged into a new virus, which is secreted by exocytosis to spread to additional cells. The NSPs have many important functions in virus pathogenesis, such as stimulating the replication enzyme (RNA polymerase), proofreading of the SARS-CoV-2 genome, suppression of interferon (IFN) signaling, blocking the translation of host RNA, and stimulating cytokine expression. SARS‐CoV‐2 triggers an immune response through proinflammatory cytokine production associated with a weak protective IFN response to viral infection [64–66].

### Endothelial cells

**Fig. 2:**
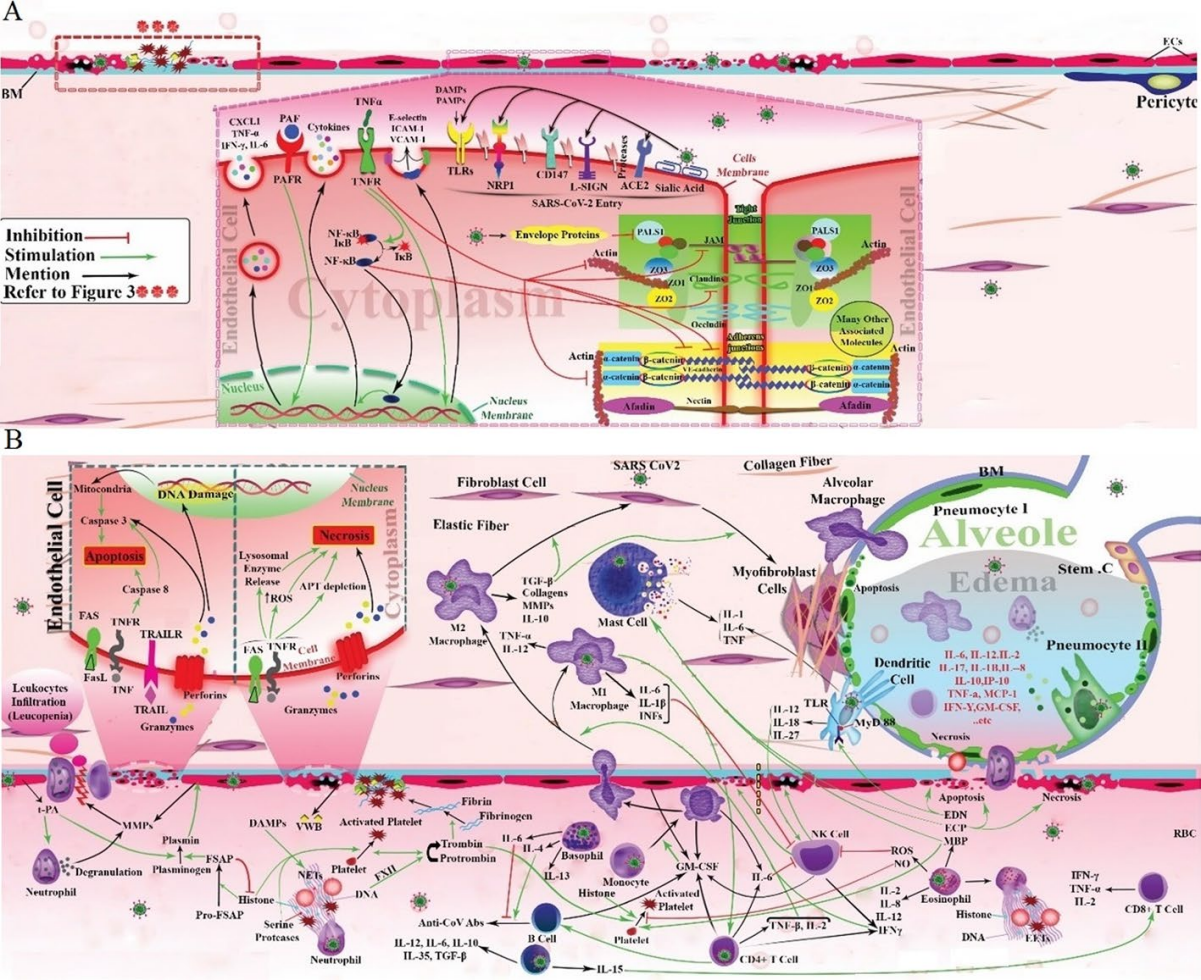
Suggested pattern for cell interactions in COVID-19, leading to pulmonary edema. In ECs, ACE2, CD147, NRP1, TLRs, L-SIGN, TMPRSS2, and sialic acid receptors may mediate SARS-CoV-2 penetration. PAF is released by a variety of cell types. ECs express PAFR. PAF/PAFR complex in ECs induces the production of cytokines such as CXCL1, TNF-α, IFN-γ, and IL-6. ECs may have TNFRs that cause surface expression of ICAM-1, E-selectin, and VCAM-1. In adherens junctions, important cytosolic partner(s) for VE-cadherin are α- and β-catenin and for nectins is afadin. TNF inhibits the expression of VE-cadherin, blocks its contact with β-catenin, affects actin cytoskeleton remodeling, and activates the NF-κB pathway, resulting in elevated expression of inflammatory genes. Some tight-junction-associated proteins include occludin, claudins, jAMs, ZO1, ZO2, ZO3, and PALS1 (**A**). SARS-CoV-2 E protein interacts with PALS. TNF disrupts claudin-5. TNF-α destroys JAM-A, claudin-4, and claudin-5. EC death occurs by apoptosis and/or necrosis. In the extrinsic pathway, TRAILR and Fas stimulation cause caspase-8 activation. Caspase-8 stimulates the caspase cascade that ultimately leads to apoptosis. FasL is released by neutrophils and lymphocytes. NK cells and cytotoxic T cells secrete perforin and granzymes that, through direct exposure to target cells, secrete perforin and granzymes, resulting in induction of apoptosis and/or necrosis. The molecular mechanism of necrosis is not clear, though it probably occurs via the release of lysosomal enzymes and generation of ROS, and in necrosis significant ATP depletion is seen. Fas and TNF stimulate both apoptosis and necrosis. ECs release t-PA, mediating the conversion of plasminogen to plasmin, and MMPs, lysing ECM. t-PA enhances neutrophil degranulation and MMP-9 secretion. Cell infiltration is facilitated by MMPs that result in leukopenia. Infected cells secrete numerous cytokines and DAMPs. DAMPs induce NETosis. NETs include DNA, histones, and enzymes such as serine protease. They are a scaffold for platelets, red blood cells (RBCs), and plasma proteins. Histones can activate pro-FSAP. FSAP, a serine protease, is a mediator of plasminogen-to-plasmin conversion. NETs activate FXII to convert prothrombin to thrombin. Thrombin converts fibrinogen to fibrin. Fibrin contributes to blood clot formation. Thrombin, NET serine proteases, and histones activate platelets. vWF is secreted by ECs and enhances platelet adhesion and aggregation. Basophils are secreted by IL-4, IL-6, and IL-13 production. They affect mature human B cells. IL-4 is correlated with the concentration of IgG antibodies, but IL-6 is inversely associated with them. Eosinophils produce NO and EETs to limit viral replication. NO inhibits platelet activation. On the other hand, EETs and MBP mediate platelet activation. Activated eosinophils secrete IL-2, IL-8, IL-12, and INF λ. EDN induces the TLR2–MyD88 signal pathway in DCs, resulting in IL-12, IL-27, and IL-18 secretion that increases NK cell activity and induces secretion of IFN-γ. IFN-γ is also secreted by NK cells. DCs also produces IL-6, significantly. ECP and EDN activate apoptotic pathways. ECP also stimulates necrosis process. In addition, increased levels of MBP and ECP stimulate the degranulation of perivascular MCs. MCs release IL-6, IL-1β, and TNF. NK cell activity can decrease by IL-6 and IL-1β. ROS can also be an inhibitor for NK cells. Eosinophils produce ROS. NK cells activate apoptosis and necrosis by secretion of FasL, TRAIL, perforin, and granzymes. B-cell and T-cell interactions lead to plasma cell generation (colonal expansion, antibody secretion) and production of either proinflammatory cytokines such as IL-12, IL-6 and IL-15 or anti-inflammatory cytokines such as IL-10, IL-35, and TGF-β by B cells. IL-12 and IL-6 provide positive feedback in B- and T-cell interactions. IL-15 enhances CD8^+^ T-cell activity. GM-CSF are produced by macrophages, B cells, T cells, NK cells, and ECs. GM-CSF stimulate the differentiation of monocytes. M1 produce proinflammatory cytokine such as IL-1β, IL-6, TNF-α, and IL-12 and INFs. M2 releases types I and III collagen, MMPs, and anti-inflammatory cytokines such as IL-10 or TGF-β. M2 can be transited into fibroblasts by TGF-β mediation, leading to pulmonary fibrosis. M1 stimulates Th cells. IFN-γ, TNF-β, and IL-2 are secreted by Th cells that activate macrophages. M1 activates NK cells by IL-1β, IFN-β, and IL-15. Alveolar macrophages release IL-1, IL-6, TNFs, and IL-8. Type 2 pneumocytes also play a major role in the formation of cytokine storms. Destruction of the air–blood barrier leads to infiltration of cells associated with alveolar epithelial cells secreting many cytokines, such as IL-1B, IL-2, IL-6, IL-7, IL-8, IL-10, IL-17, TNF, etc., out of control, resulting in further and further injury. Finally, lung edema and pulmonary failure occurs (**B**). COVID-19, coronavirus disease 2019; ACE2, angiotensin-converting enzyme 2; ECs, endothelial cells; CD147, cluster of differentiation 147; TLRs, Toll-like receptors; NRP1, neuropilin-1; L-SIGN, homolog dendritic cell-specific intercellular adhesion molecule-3-grabbing non-integrin related; serine 2; TMPRSS2, transmembrane protease, SARS-CoV-2, severe acute respiratory syndrome coronavirus 2; PAFR, platelet-activating factor receptor; PAF, platelet-activating factor; TNF, tumor necrosis factor; IL, interleukin; IFN, interferon; TNFRs, tumor necrosis factor receptor; ICAM-1, intercellular adhesion molecule-1; VCAM-1, vascular cell adhesion molecule-1; VE-cadherin, vascular endothelial cadherin; ZO, zonula occludens; JAMs, junctional adhesion molecules; E protein, envelope protein; PALS1, protein associated with LIN7 1, MAGUK family member; TRAIL, TNF-related apoptosis-inducing ligand; t-PA, tissue plasminogen activator; NK cells, natural killer cells; NF-κB, nuclear factor kappa-light-chain-enhancer of activated B cells; MMPs, matrix metalloproteinases; ECM, extracellular matrix; FSAP, factor VII activating protease; DAMPs, damage-associated molecular pattern; NETs, neutrophil extracellular traps; NO, nitric oxide; vWF, von Willebrand factor; EETs, eosinophil extracellular traps; EDN, eosinophil-derived neurotoxin; MBP, major basic protein; MyD88, myeloid differentiation factor 88; ECP, eosinophil cationic protein; DCs, dendritic cells; MCs, mast cells; GM-CSF, granulocyte–macrophage colony-stimulating factor; TGF-β, transforming growth factor beta; M1, type 1 macrophages; M2, type 2 macrophages; Th cells, T-helper cells

As shown in Fig. 2, endothelial cells ACE2 [20], transmembrane serine protease 2 (TMPRSS2) [21], sialic acid receptors (a surface adhesion molecule) [22], extracellular matrix metalloproteinase inducer (CD147, or basigin), TLR2, TLR4, TLR5, and TLR9 [23], and NRP1 [24] are all reportedly implicated in SARS-CoV-2 entry. Moreover, it is reported that cathepsin B and L are also essential entry factors in COVID-19. L-SIGN is reportedly expressed in type II human alveolar cells, and pulmonary endothelial cells also mediate SARS-CoV-2 entry [67].

When the SARS-CoV-2 enters endothelial cells, it adversely affects these cells. Moreover, endothelial cells can be affected by molecules produced by other cells fighting against the virus. These molecules can bind to their receptors expressed on the endothelial cell surface. Platelet-activating factor (PAF) is one molecule released by a variety of cell types and tissues, but the main source of PAF is leukocytes. PAF mediates the inflammation process. Platelet-activating factor receptor (PAFR) is thought to be formed on the cytoplasmic or nuclear membrane of various cells, in particular endothelial cells, platelets, and leukocytes. The PAF/PAFR complex in endothelial cells may induce increased vascular permeability, hypotension, and expression of cytokines such as CXCL1, interleukin 6 (IL6), tumor necrosis factor-alpha (TNF-α), and IFN-γ [68, 69].

TNF-α is another signaling molecule that may affect endothelial cells in COVID-19. Infection by SARS-CoV-2 strongly induces the secretion of TNF-α, which has an essential role in the pathology of acute lung injury. TNF signaling and the production of two TNF receptors (TNFRs, p55 and p75) on the pulmonary endothelium have not been clearly demonstrated in humans. One study reported that the two receptors, predominantly p55, are produced in pulmonary endothelial cells in mice. Reportedly, the administration of intravenous TNF led to surface expression of E-selectin, VCAM-1, and ICAM-1 [70, 71]. Moreover, studies have reported that TNF can affect cell–cell junctions. SARS-CoV-2 has many effects on cell junctions, especially in endothelial and epithelial cells. Adherens junctions are specific intercellular adhesive structures mainly formed by cadherin and nectin adhesion receptors. They are essential for organization and flexibility of cell-to-cell attachment. Adherens junctions in endothelial cells are complexes containing vascular endothelial cadherin (VE-cadherin) and nectins. There are many cytosolic partners that interact with them, such as α- and β-catenin for VE-cadherin, and afadin for nectins [72, 73].

TNF can inhibit the expression of VE-cadherin, and also increase the tyrosine phosphorylation of VE-cadherin that blocks its binding with β-catenin, thus resulting in nonfunctional junctions [74, 75]. Furthermore, TNF can affect actin cytoskeleton remodeling, VE-cadherin cytosolic domains, and β-catenin interactions. It has also been reported that TNF can induce caspase activation, which destroys intercellular junctions. In addition to cell junctions, members of the TNF family can activate the nuclear factor kappa-light-chain-enhancer of activated B cells (NF-κB) pathway, resulting in increased proinflammatory gene transcription, cell proliferation, and differentiation [76, 77].

Tight junctions between endothelial cells include transmembrane proteins such as MARVEL domain proteins, e.g., occludin, junctional adhesion molecules (jAMs), and claudins [66–68]; adaptor proteins; cytoskeletal linkers; and polarity proteins, e.g., zonula occludens (ZO1, ZO2, and ZO3) proteins and protein associated with Lin-1 1 (PALS1) [69–71]. Atypical protein kinase C (aPKC) is another component of the signaling pathway related to tight junctions [72].

Capillary leakage can occur in severe infections or in sepsis, due to disruption of tight junctions between endothelial cells. It has been suggested that hijacking of PALS1, a tight-junction-associated protein, by the SARS-CoV E protein has a central role in lung epithelial disruption in these patients [78]. Studies have reported that PALS1 is also expressed in endothelial cells. Researchers have proposed that the SARS-CoV-2 E protein can interact strongly with PALS1, thereby disrupting the epithelial barrier and leading to severe inflammation [79, 80].

Moreover, it has been shown that TNF can disrupt claudin-5 in tight junctions by an NF-κB-dependent process, and activate kinase cascades in human dermal endothelial cells. However, so far, no study has been done on pulmonary endothelium [81]. Another study has reported that TNF can lead to barrier destruction and mislocalization of JAM-A, claudin-4, and claudin-5, which are involved in tight junction structure [82].

In addition to disruption of endothelial cell junctions during inflammation and viral infections, it is known that endothelial cell death may occur following SARS-CoV-2 infection by apoptosis and/or necrosis, resulting in capillary leakage, lung failure, and MODS. Programmed cell death apoptosis can be triggered by two signaling pathways: extrinsic (death receptor-dependent) or intrinsic (mitochondrially mediated). Extrinsic pathway-activated caspase-8 can also activate the intrinsic pathway. Activated caspase-8 can stimulate caspase-3 directly or by promoting mitochondrial cytochrome C release [83, 84].

In the extrinsic pathway, the death receptors Fas and TNF receptor are activated, resulting in caspase-8 activation. Caspase-8 stimulates other caspase cascades that ultimately lead to apoptosis [85]. Fas and Fas ligand (FasL) are an important system that mediates extrinsic pathway apoptosis. FasL is released by neutrophils and lymphocytes, especially under inflammatory conditions. Fas is a surface receptor expressed in many types of respiratory cells, including endothelial, alveolar, and bronchial epithelial cells and macrophages. The Fas/FasL system is hyperactivated in patients with ARDS, and it has been reported that activation of this complex in the lungs can lead to the release of TNF-α, transforming growth factor-β1 (TGF-β1), and other proinflammatory cytokines leading to epithelial apoptosis [86]. Another component of the death receptor ligand family is the transmembrane protein TNF-related apoptosis-inducing ligand (TRAIL) [87].

It has been established that cytotoxic T lymphocytes and natural killer (NK) cells are involved in the immune surveillance of virus-infected or virally transformed cells [88]. These effector cells secrete a membrane-disrupting protein called perforin, and granzymes, structurally related serine proteases, acting as cytotoxic mediators, along with proinflammatory cytokines (IFN-γ and TNFα) [89]. Perforin and granzymes are released by exocytosis to induce apoptosis of the infected or transformed cells [90]. In the granule-exocytosis pathway of lymphocyte-mediated cell death, activated lymphocytes secrete both perforin and granzymes. Monomers of perforin may act together to form the polymeric pore structure in the plasma membrane, causing osmotic cytolysis, a type of necrosis. The pathways responsible for granzyme-induced cell death are unclear. It has been suggested that granzymes penetrate the target cell to begin an internal disintegration pathway, which ultimately causes DNA fragmentation and apoptosis or cell lysis [91, 92]. Moreover, the accumulation of cell death receptors such as Fas may occur. In this pathway, the Fas expressed on the target cells binds to the FasL expressed on the killer-cell membrane. This Fas–FasL complex then promotes caspase-dependent apoptosis [93].

The molecular mechanisms of necrosis are not completely clear. It has been suggested that necrosis probably occurs by the release of lysosomal enzymes, generation of ROS, and activation of phospholipases [94]. Elevated levels of ROS can destroy the mitochondrial respiratory chain, inhibiting the transfer of electrons to O_2_, thus further increasing ROS production by a positive feedback loop. A large number of damaged mitochondria will lead to necrosis. In cell death mediated by lysosomal damage, lysosomal enzymes are released into the cytosol, resulting in necrosis [95]. Some studies have reported that FasL and TNF can stimulate both apoptotic and necrotic pathways. Interestingly, although a significant depletion of intracellular ATP has been seen in apoptosis, in necrosis the levels of ATP remained at the normal concentration seen in healthy cells [96, 97].

### Platelets

Studies have shown that coagulopathy is a common symptom in patients with severe COVID-19. This could be a result of excessive inflammation. Coagulopathy is characterized by dysregulated coagulation parameters, lower platelet count, increased microthrombi, elevated D‐dimer, etc. It is reported that patients with COVID‐19 who have lower platelet counts and disseminated intravascular coagulation have a poor prognosis and increased mortality [27, 28]. In many viral infections, platelets can be activated, and are involved in inflammation and thrombotic processes. The low platelet level in some patients with COVID-19 could be due to increased consumption and thrombus formation by massively activated platelets [98, 99].

Platelets contain alpha granules (containing VWF, fibrinogen, VEGF, MMP2 etc.), delta granules (containing serotonin, ADP, ATP, polyphosphates etc.), T granules (responsible for TLR organization), and lysosomal granules. Vesicle-associated membrane proteins (VAMPs) may mediate platelet endocytosis. Fibrinogen is imported into platelet α-granules and is then fused with vWF [100–103].

**Fig. 3:**
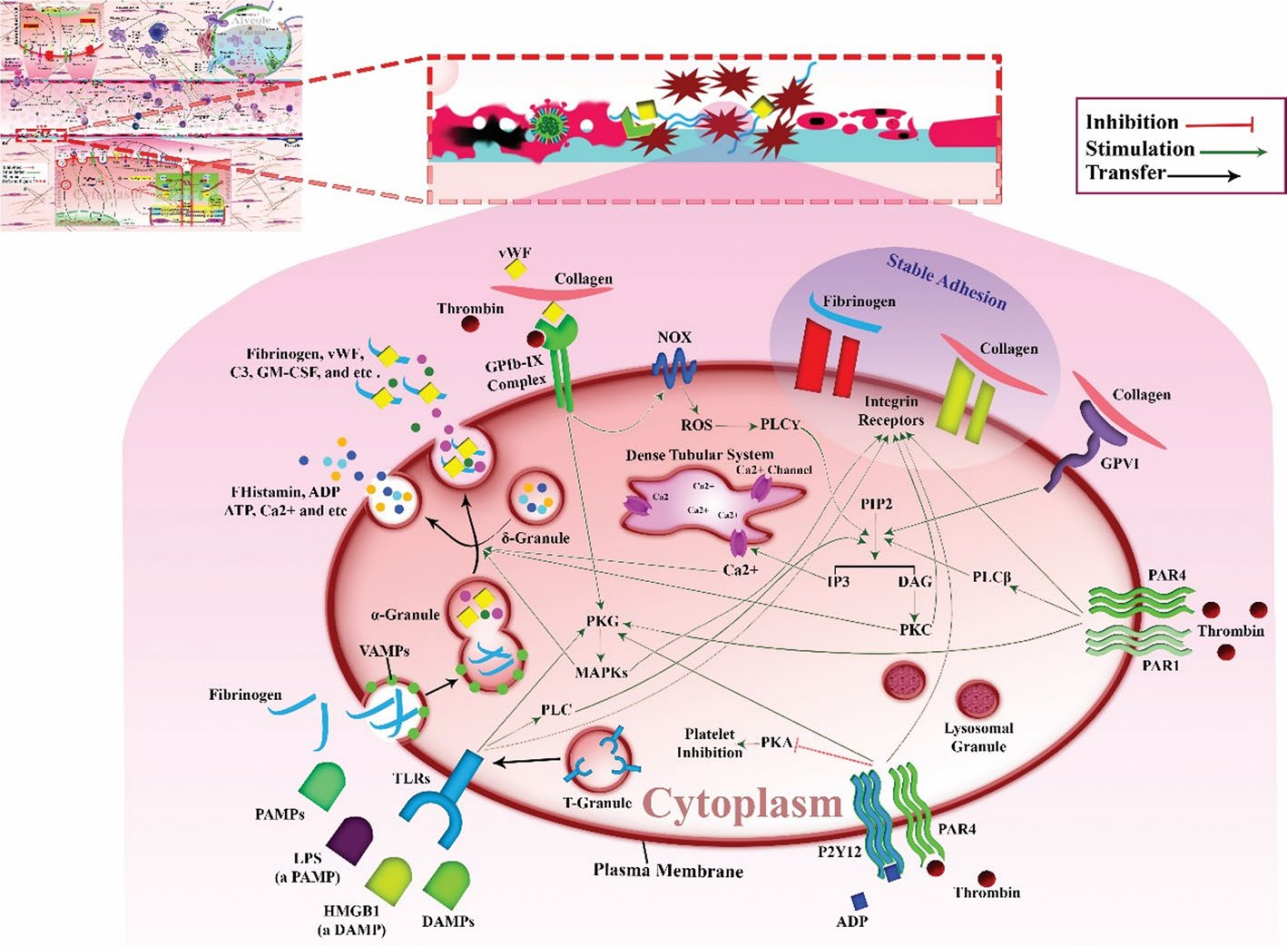
Platelet receptor/signaling and COVID-19. Low platelet level in some patients with COVID-19 can be due to massively activated platelets. Platelets contain alpha, delta, T, and lysosomal granules. VAMPs may mediate platelet endocytosis. Fibrinogen normally imports into the platelet α-granules and is fused with vWF. Platelets have various receptors: integrins; GP Ib/IX/V; TLRs; thrombin receptors of PAR-1 and PAR-4; ADP receptor of P2Y12; GPVI. In viral infections, TLRs are activated that cause the secretion of complement C3 from alpha granules in platelets. They also release GM-CSF. Complement C3 and GM-CSF stimulate NETosis. SARS-CoV-2 pathogenesis indirectly induces an enhanced capability of VWF to bind to its receptors on platelets. VWF binds to GPIb-IX-induced transient platelet adhesion. Thrombin is another ligand for GPIb-IX. It stimulates the PKG and MAPK pathway and, ultimately, granule secretion. GPVI is known as a collagen receptor. In this pathway, PIP2 hydrolyzes into DAG and IP_3_ by PLC2 mediation. DAG and IP_3_ are known as secondary messengers. DAG activates PKC isoforms. PKCs are involved in integrin activation and platelet granule secretion. IP_3_ increases calcium concentration in cytosol of cells by affecting the dense tubular system channel. Calcium elevation is also required for stable platelet adhesion, granule secretion, procoagulant activity, and clot retraction and collectively almost all platelet functions. PAMPs and DAMPs (such as HMGB1) bind to PRRs, such as TLRs. They prompt platelet activation. TLRs activate PLC2. They also activate the PKG pathway. Minimally three thrombin receptors on human platelet surface have been defined, i.e., GPIb-IX, PAR1, and PAR4. GPIb-IX signaling plays a pivotal role in the assembly of NOX subunits and ROS production. It can also activate PLC through a ROS-mediated signaling pathway. Then, PLC activation leads to DAG and IP_3_ formation, which has already been described. PAR1 and PAR4 also activate PLC isozymes and have an effect on the PKG and MAPK pathway. Integrins are also important platelet molecules involved in platelet activity, adhesion, and aggregation, including collagen, fibronectin, etc. They are substantial for the stable adhesion and aggregation of platelets. COVID-19, coronavirus disease 2019; GP Ib/IX/V, glycoprotein, Ib/IX/V; TLRs, Toll-like receptors; GM-CSF, granulocyte–macrophage colony-stimulating factor; C3, complement component 3; SARS-CoV-2, severe acute respiratory syndrome coronavirus 2; PKG, protein kinase G; vWF, von Willebrand factor; MAPK, mitogen-activated protein kinase; PIP2, phosphatidylinositol 4,5-bisphosphate; DAG, 1,2-diacylglycerol; IP_3_, 1,4,5-trisphosphate; PKC, protein kinase C; PLC2, phospholipase C-2; DAMPs, damage-associated molecular patterns; PAMPs, pathogen-associated molecular patterns; NOX, nicotinamide adenine dinucleotide phosphate oxidase; PRRs, pattern recognition receptors

Platelets have a variety of transmembrane receptors (Fig. 3), including integrins (aIIb3, a5b1, a2b1, aVb3, a6b1); leucine-rich repeat receptors (LRR receptors); Toll-like receptors (TLRs); glycoprotein Ib/IX/V (GP Ib/IX/V); G-protein-coupled seven transmembrane receptors (GPCR) including thrombin receptors PAR-1 and PAR-4; ADP receptors P2Y1 and P2Y12; and TxA2 receptors TP-a and TP-b. Other proteins belong to the immunoglobulin superfamily, including GP VI and FcgRIIA, C-type lectin receptors (P-selectin), and tyrosine kinase receptors including Gas-6, thrombopoietin receptor, ephrins, and Eph kinases. Other receptors include CD63, P-selectin ligand 1, CD36, TNF receptors, etc. [26].

Collectively, it has been suggested that platelets may have an important role in the pathogenesis of COVID‐19 infection. Platelets can internalize the virus, resulting in TLR7 activation and the secretion of complement component 3 (C3) from their alpha granules. C3 stimulates the process of NETosis, which traps any viruses missed by platelets [104, 105]. In NETosis, the nuclear material of neutrophils is released into the extracellular space. These structures form a meshwork of chromatin called neutrophil extracellular traps (NETs). In this meshwork, the DNA is coiled around histones and enzymes derived from neutrophil cytoplasmic granules. It has been suggested that NETosis is a type antimicrobial defense mediated by neutrophils. These structures also act as a scaffold for the attachment of RBCs, platelets, and plasma proteins to form a trap for any virus missed by platelets [106, 107].

In response to unknown signals from neutrophils, platelets release granulocyte–macrophage colony-stimulating factor (GM-CSF) to regulate NETosis. Interestingly, there are reports of increased levels of both GM-CSF and C3 in COVID-19 infections [104, 105].

COVID-19-related endothelial cell death can be a result of direct damage by the virus, or indirect damage by cytokines leading to thrombosis, particularly in small vessels with low blood flow. The exposed subendothelial matrix following endothelial cell death causes accumulation and activation of platelets intended to repair the damage [29, 108]. When further endothelial cells are destroyed, more platelets are activated that lead to microthrombus formation. It is known that healthy endothelium secretes prostaglandins, nitric oxide (NO), and adenosine to control the extent of thrombus formation by dissolution, and to regulate platelet activation [109, 110].

Platelets can also be affected by inflammatory cytokines and high antibody concentrations, which can lead to increased thrombosis. The levels of IL-6 and IL-1β are increased in patients with COVID-19, and it has been reported that IL-6 and IL-1β are involved in platelet hyperactivation [111–114].

Infected endothelium can also lead to the secretion of tissue factor (TF), a well-known coagulation cascade activator. TF leads to platelet aggregation for clot formation via the stimulation of thrombin and the generation of fibrin fibers. Even if anticoagulant therapy is administered, this may lead to deep vein thrombosis and pulmonary embolism. Following the activation of platelets, they produce functionally active TF [99, 115]. The effect of cytokines on platelets and endothelial cells in COVID-19 is not fully understood [116].

It has been found that SARS-CoV-2 infection indirectly induces an increased ability of von Willebrand factor (vWF) to bind to its receptors on platelets [102]. The vWF on the endothelial surface interacts with its receptor (GP Ib–IX–V complex) on platelets. The cytoplasmic GPIb domain underlying the plasma membrane of platelets binds to the actin filaments. It is important to protect the shape and structure of platelet membranes, which is essential for platelet adhesion [117, 118]. The binding of vWF to GPIb-IX induces transient platelet adhesion. Platelet signals then activate integrins resulting in stable adhesion. Thrombin is another ligand for GPIb-IX, which is involved in low-dose thrombin activation of platelets, activation of protein kinase C (PKG), and activation of the mitogen-activated protein kinase (MAPK) (ERK1/2 and p38) pathway, and finally secretion of platelet granules [119–122].

GPVI is known as a collagen receptor, and plays a key role in collagen-mediated platelet activation. In this pathway, phosphatidylinositol 4,5-bisphosphate (PIP2) is hydrolyzed to produce inositol 1,4,5-trisphosphate (IP_3_) and 1,2-diacylglycerol (DAG) by phospholipase C-2 (PLC2) activity. DAG and IP_3_ are known as cellular secondary messengers. DAG activates PKC isoforms, which are involved in the activation of integrins and the secretion of platelet granules [123–125].

IP_3_ increases the calcium concentration (Ca^2+^) in the cytosol by triggering the release from the dense tubular system. This depletion of intracellular Ca^2+^ storage activates store-operated calcium entry resulting in sustained increases in intracellular calcium [126].

An increase in intracellular calcium is crucial for several intracellular signaling processes. PKC, calpain, and calmodulin are all proteins that are activated by calcium. Moreover, integrin signaling often depends on Ca^2+^ levels. Ca^2+^ elevation is also required for stable platelet adhesion, granule secretion, procoagulant activity, clot retraction, and almost all platelet functions [125].

Pathogen-associated molecular patterns (PAMPs) are defined as molecules secreted by microorganisms. These can be DNA, proteins, RNA, lipopolysaccharides (LPS), and glycoproteins. Moreover, damage-associated molecular patterns (DAMPs) are also secreted from infected or damaged tissue. DAMPs can be proteins, DNA fragments, or lipid oxidation products. PAMPs and DAMPs can bind to pattern recognition receptors (PRRs), such as nucleotide-binding oligomerization domain (NOD)-like receptors, TLRs, and the advanced glycation end-product (RAGE) receptor. It has been reported that TLRs and NODs can trigger platelet activation. In platelets, the collagen receptor GPVI can activate PLC2, resulting in the formation of DAG and IP3, which can trigger PKC and stimulate Ca^2+^ liberation, thus affecting platelet function [127–129].

TLR4 is the most important platelet receptor for LPS. It triggers signals that finally result in granule secretion. It has been shown that the activation of the cGMP–PKG pathway mediated by MyD88 has an important role in TLR4-dependent platelet activation. TLR4 can also recognize high-mobility group box 1 (HMGB1), a DAMP protein that activates platelets and triggers granule secretion. HMGB1 is a DNA-binding protein produced by macrophages and monocytes during infection and inflammation [25, 130, 131].

Thrombin is another ligand binding to platelet receptors. Up to now, at least three thrombin receptors have been identified on the surface of human platelets: GPIb-IX, PAR1, and PAR4 [132, 133]. The presence of these three different receptors can increase the sensitivity of platelets to low thrombin levels, and is pivotal to thrombosis formation [134]. It has been reported that GPIb-IX signaling activated by thrombin and vWF mediates platelet activation by a member of the Src kinase family (Lyn or possibly Src) interacting with the Rac1 signaling pathway [135].

Rac1 is a GTPase with an important role in the assembly of subunits of nicotinamide adenine dinucleotide phosphate oxidase (NOX), as well as in ROS production. It also has been suggested that thrombin can activate PLC through a ROS-mediated signaling pathway. Then PLC activation leads to DAG and IP3 production, as described above [135, 136].

PAR1 and PAR4 can also activate PLC isozymes to produce PIP2, which is hydrolyzed into IP3 and DAG, which can then promote the production of thromboxane (TXA). It has been reported that high levels of TXA2 can induce granule secretion in platelets. The activation of the Src family kinase–Rac1 signaling pathway is also involved in PAR1/PAR4-dependent platelet activation and granule secretion, by the mitogen-activated protein kinase (MAPK) pathway [136–139].

Integrins are also important molecules involved in platelet activation, accumulation, and adhesion. Integrins act as receptors for collagen, fibronectin, laminin, and vitronectin. The integrin α_IIb_β_3_ has been reported to be the most important and abundant member of the family. It is a receptor for fibrinogen, vWF, and several matrix adhesion proteins. Integrin α_IIb_β_3_ is needed for stable adhesion and aggregation of platelets [140, 141].

Collectively, calcium elevation, PKC signaling, SFK signaling, and MAPK signaling, among others, are the platelet signaling pathways involved in granule secretion. However, the complete details of these mechanisms are not clear. It is known that coagulopathy due to SARS-CoV-2 infection has a poor prognosis. Therefore, we propose that further studies focusing on platelets and their receptors in COVID-19 are needed.

### Respiratory failure: cellular interactions in the lungs

SARS-CoV-2 infection of lung endothelial cells, which might occur from the luminal or the alveolar interstitial side, triggers endothelial release of cytokines, which cause increased capillary permeability, thereby allowing adhesion and extravasation of neutrophils and monocytes into the alveolar interstitial space. Stimulated by PAMPs and DAMPs, neutrophils and macrophages secrete a multitude of cytokines, procoagulants, and complement, which promote viral attack and clearance but induces further vascular injury enhancing the risk for thrombosis. Several factors might contribute to the prothrombotic environment, thereby promoting intravascular thrombus formation: (a) neutrophils mediate secretion of NETs (neutrophil extracellular traps), and complement enhances platelet aggregation; (b) cytokines trigger secretion of tissue factor (TF) by endothelial cells, and macrophages stimulate coagulation cascade and increases in fibrin clot formation; (c) endothelial damage decreases secretion of anticothrombotic mediators, such as antithrombin (AT) and TF pathway inhibitor (TFPI); (d) lung residential megakaryocytes produce locally available platelets for aggregation; (e) the angiotensin-converting enzyme (ACE)/Ang II (angiotensin II)/AT1 receptor axis is overactivated owing to virus-induced ACE2 downregulation increasing production of PAI1 (plasminogen activator inhibitor 1), reducing plasmin activation and fibrinolysis [142]. Several factors that might contribute to respiratory failure in COVID-19 disease are summarized as follows:

### MMP-9-related respiratory failure in COVID-19 disease

A schematic illustration of the cellular interactions in the lungs is shown in Fig. 2. The endothelial cell layer is lined by a glycocalyx layer, and can release tissue plasminogen activator (t-PA) to inhibit platelet binding and activation of the coagulation cascade. t-PA mediates the conversion of plasminogen into plasmin, with an important role in fibrinolysis. It has been reported that SARS-CoV-2-infected endothelial cells can secrete t-PA. High t-PA concentrations present in COVID‑19 samples can carry out spontaneous fibrinolysis [143, 144]. It is known that t-PA can enhance neutrophil degranulation, while t-PA administration can increase blood levels of matrix metalloproteinase (MMP)‐9 (gelatinase B) [145]. It should be mentioned that MMPs are proteolytic enzymes that degrade extracellular matrix (ECM) components, such as collagen, proteoglycans, and elastin. It has been reported that endothelial cells secretes several MMPs under pathological conditions [146].

MMP-9 is released by neutrophils, monocytes, macrophages, eosinophils, and other inflammatory cells. In acute inflammation, neutrophils undergo chemoattraction to accumulate at the injury site. MMP-9 is released from neutrophils by degranulation and then degrades the basement membrane collagen (especially type IV collagen). Inflammatory cell infiltration is facilitated by MMPs, which then results in leukopenia [147–149].

### Role of DAMPs in SARS CoV-2-induced respiratory failure

Widespread endothelial dysfunction caused by direct viral infection or immune response results in the secretion of numerous cytokines and DAMPs. DAMPs can directly induce NETosis following influenza virus infection [150, 151]. NETs are made up of DNA, histones, and enzymes such as serine proteases [106, 107]. The released histones can activate pro-factor VII activating protease (pro-FSAP) to produce factor VII activating protease (FSAP). FSAP contributes to the coagulation and fibrinolysis cascades, by indirectly mediating plasminogen activation. Interestingly FSAP can also degrade histones [152].

NETs can also stimulate FXII activation, and FXIIa is able to directly convert prothrombin to thrombin, which is a key factor in coagulation. Thrombin causes fibrinogen conversion to fibrin, which is amplified by factor XIII activation. Then, the soluble fibrin monomers interact with each other to produce a cross-linked network of long fibrin threads that trap the platelets. Then a spongy mass builds up, hardens, and contracts to form the final blood clot. The coagulation cascade is important both in normal circulatory conditions and in pathological reactions [152, 153]. Moreover, NET serine proteases and histones may also play a role in platelet activation [106, 154, 155].

### The role of von Willebrand factor and granulocyte subsets in SARS CoV-2-induced respiratory failure

Von Willebrand factor (vWF) is another factor involved in SARS-CoV-2 coagulopathy. It is secreted by endothelial cells and megakaryocytes. Increased vWF antigen concentrations in plasma have been measured in COVID-19 infections [155, 156]. Reportedly, the increased concentration of vWF enhanced the adhesion of platelets onto the collagen surface, and increased the aggregation of platelets. vWF could be a therapeutic target in patients with thrombocytopenia [157].

On the basis of some studies, basophil and eosinophil counts, as well as granulocyte subsets, are altered in severe COVID-19 infection. These cells play a pivotal role in the antiviral response and immunopathology in COVID-19. Basophils are decreased during the acute phase, while the numbers recover during the COVID-19 recovery period. Basophils can enhance B-cell responses during the humoral immune response triggered by IL-4 or IL-6 production. Moreover, it has been shown that basophil count is significantly correlated with the concentration of IgG antibodies against SARS CoV-2 [157]. However, the level of IL-6 is inversely correlated with antibody production [158]. It was also reported that basophils can secrete IL-13 following activation, and IL-13 receptors are expressed on mature human B cells, so basophils may contribute to humoral immune responses and inflammatory allergic reactions [159].

Notch signaling is a juxtacrine signaling pathway, mediated by receptor–ligand interactions between neighboring cells. The notch signaling pathway is dysregulated in many pathological conditions, especially inflammation [160–162]. In experimental animal models, the pharmacological blocking of notch signaling has been reported to improve inflammatory conditions [163]. In vitro, it was shown that cytokines can affect notch signaling in basophils, and inhibition of this signaling pathway reduced basophil cytokine production. It was also reported that the notch signaling pathway is involved in the expression of cytokine genes in activated macrophages during inflammation [164]. Notch signaling is also important for peripheral T-cell differentiation and activation [165].

The overexpression of Jagged (a notch ligand) in professional antigen-presenting cells (APCs) has been reported [166]. Although there is not enough evidence for basophils to function as antigen-presenting cells, it is known that B cells are APCs, and can activate T cells [167]. Moreover, basophils can enhance B-cell responses [158].

Because COVID-19 is an inflammatory disease, it may also affect the notch signaling pathway. As a result, basophil cytokine production and basophil-induced B-cell activity would be impaired, affecting both cellular and humeral immunity. This is still a hypothesis, and further studies are needed to confirm the role of the notch signaling pathway in COVID-19.

Eosinophils are another granulocyte subset that contribute to viral infections by producing NO, increasing TLR7, and can also produce eosinophil extracellular traps (EETs, similar to NETs) to limit viral replication [168]. It was also reported that eosinophils are involved in the immune response against rhinovirus infections by stimulating T cells [169]. NO is a major endogenous vasodilator that maintains blood pressure within the normal range and also blocks platelet activation [168, 170]. It was reported that the increase in blood eosinophils found in patients with eosinophilic asthma exerted no protective influence on COVID-19 [171]. A variety of eosinophil functions in patients has been reported. Eosinophil EETosis and release of major basic protein (MBP) could contribute to coagulopathy, while in blood vessels, eosinophils could mediate platelet activation and thrombus formation [172].

It has been reported that activated eosinophils can secrete cytokines, such as IL-2 [172], IL-8 [173], IL-12 [174], INF λ [175], etc. Eosinophil degranulation occurs in inflammatory conditions and virus infections. The important components of eosinophil granules include eosinophil-derived neurotoxin (EDN), major basic protein (MBP), eosinophil peroxidase, and eosinophil cationic protein (ECP) [176].

EDN can induce the activation of the dendritic cell TLR2–MyD88 signaling pathway in response to microbial and viral infections [176]. Both macrophages and DCs can secrete IL-12, IL-27, and IL-18. IL-18 has a significant role in ARDS [176–178]. IL-12 acts on activated NK and T cells expressing the IL-12 receptor. IL-12, IL-27, IL-15, IL-18, and type I IFN cal all increase the activity of NK cells and induce the expression of IFN-γ. IFN-γ can activate macrophages to destroy pathogens. Moreover, it was shown that IL-12 can trigger the differentiation of T-helper 1 cells. Pulmonary DCs also produce IL-6 at a significantly higher level than other DCs. Some studies have reported that IL-6 is the most important single cytokine in the cytokine storm seen in patients with COVID-19 [179–181].

ECP and EDN have both been reported to activate apoptotic pathways in infected cells. Moreover, ECP is strongly associated with tissue necrosis in different organs. In addition, higher concentrations of MBP and ECP occur in patients with eosinophilic asthma. They could contribute to many biological processes, such as the stimulation of perivascular mast cells (MCs) resulting in microvascular permeability [176, 182].

Various proinflammatory cytokines are released in patients with COVID-19, especially IL-6. It has been reported that IL-6 is a pivotal mediator of lung damage. Mast cells (MCs) have been found to be important sources of IL-6, IL-1β, VEGF, and TNF, and they play a crucial role in several pulmonary diseases [183].

### The role of activated cells of the immune system in SARS CoV-2-induced respiratory failure

NK cells have also been identified as important effector cells for the innate immune response. NK cells are involved in the early defense against viral infections and to deal with malignant transformation. Studies have shown that ROS can act as an inhibitor of NK cells [184, 185]. ROS can produced by eosinophils, and their ROS production may be stimulated by immunoglobulins (Igs), adhesion molecules, and cytokine signaling. ROS are essential for optimal defense against viruses, but they may also contribute to viral pathogenesis and host cell destruction. NK cell cytotoxic activity can by decreased by IL-6. Studies have reported that the inhibition of IL-6 and IL-1 can increase the expression of perforin, granzyme, and IFN [186–190].

However, contrary to expectations, some studies found that the percentage of NK cells was dramatically higher in patients suffering from COVID-19 infection admitted to the intensive care unit (ICU) [191]. NK cells can release FasL and TRAIL death ligands, which bind to death receptors on target cells and trigger the extrinsic apoptosis pathway. NK cells can eliminate target cells via both apoptosis and necrosis. NK cells also secrete several cytokines, and are the major source of IFN-γ in the early stage of viral infection, where they coordinate the adaptive and innate immune responses [192, 193].

Adaptive immunity depends on lymphocytes, CD8^+^ and CD4^+^ T cells, and B cells. Plasma cells are antibody-producing cells that are derived from B cells. They govern the humoral response to foreign antigens by producing Ag-specific B cells and CD4^+^ T-helper cell interactions. In COVID-19 infections, plasma cells secrete anti-SARS antibodies [194].

B cells can be stimulated by either anti-inflammatory or proinflammatory cytokines. After contact with T cells, they produce proinflammatory cytokines such as IL-12, IL-15, and IL-6. IL-6 and IL-12 can form a positive feedback loop between B and T cells [195, 196]. It was reported that IL-15 secretion could enhance the migration and cytotoxicity of CD8^+^ T cells in the brains of MS animal models. It has been demonstrated that regulatory B cells can produce anti-inflammatory cytokines, such as IL-10, TGF-β, and IL-35. Pregnant women show high concentrations of immunosuppressive cytokines (IL-10, TGF-β, and IL-35) and chemokines (CCL22 and CCR4) [197–199].

B cells are also important in the initiation of an inflammatory response and GM-CSF secretion. In addition, GM-CSF can be expressed by various cells, including macrophages, T cells, endothelial cells, and NK cells. It was reported that the number of GM-CSF-expressing cells was higher in patients with COVID-19 in the ICU [191, 200, 201].

GM-CSF can stimulate the differentiation of monocytes into macrophages or into DCs [202]. Both alveolar and interstitial macrophages can be categorized as either M1-like macrophages (M1, classically activated) or M2-like macrophages (M2, alternatively activated) [203]. The M1 and M2 phenotypes have different cytokine profiles and cellular functions. M1 has antimicrobial activity and produces proinflammatory cytokines, such as IL-1β, TNF-α, IL-12, IFNs, and IL-6. On the other hand, M2 contributes to tissue maintenance, repair, and angiogenesis, and secretes anti-inflammatory cytokines. In viral infections such as COVID-19, both types of macrophage are involved. M1 can recognize and destroy viruses in various families, such as Coronaviridae, and promote an inflammatory response. On the other hand M2 plays a role in tissue repair and resolution of lung damage. Therefore, these two phenotypes must be balanced for optimal antiviral immune response, resulting in pathogen elimination without tissue injury [204].

It has also been observed that, after the induction of inflammation, M1 can be stimulated to switch to the M2 phenotype. This conversion is marked by a lower production of inflammatory cytokines and an increased secretion of anti-inflammatory cytokines (IL-10 or TGF-β). The conversion is involved in the amelioration of inflammation and protection of tissue integrity [205].

M2 produces various ECM components (type I and III collagens, and MMPs). It was reported that M2-like macrophages can be converted into collagen-producing fibroblasts by the effects of TGF-β [206]. Pulmonary fibrosis after COVID-19 pneumonia is a common complication in COVID-19 survivors [207].

M1 can stimulate activity of T-helper type 1 cells and promote cell-mediated immune responses. Th1 cells secrete IFN-γ, TNF-β, and IL-2, and can activate macrophages to increase phagocyte-dependent responses [208]. Macrophages and NK cells interact with each other, as an important antimicrobial and antitumor defense mechanism. M1 can increase NK cell cytotoxicity by affecting the IL-1β, IFN-β, and IL-15 pathways [209]. Alveolar macrophages are resident cells within the lung alveoli that act as a major early defense against respiratory infections. They release various anti-inflammatory and proinflammatory cytokines. Alveolar macrophages were found to release IL-1 (low amount), IL-6, TNF (moderate amount), and IL-8 (high amount) [210].

Moreover, in the alveolar epithelium, type 2 pneumocytes play a role in the cytokine storm and respiratory failure. The pneumonocytes produce, release, and reuptake the pulmonary surfactant and surfactant proteins A and D, control the amount of alveolar fluid, and produce some immunomodulatory proteins [211, 212].

## Conclusion

In COVID-19 infection, virus entry can be mediated by a variety of receptors and proteins. These receptors may be useful in therapeutic approaches, and more studies should be performed. On the other hand, after the destruction of the air–blood barrier caused by apoptosis or necrosis of epithelial and endothelial cells, and disruption of the basement membrane, a variety of cells enter the alveolar space in an abnormal manner, depending on the severity of the damage. Moreover, the damaged endothelial cells are involved in the coagulopathy process. It is suggested that clot formation may restrict blood flow and could lead to respiratory failure. Platelets are another important cell type in this process. Platelets have many surface receptors involved in coagulopathy and inflammation, especially by activating kinase cascades leading to granule secretion. Cytokines are the main mediator in these events. It has been shown that infiltrated inflammatory cells and lung epithelial cells are able to secrete cytokines (IL-1β, IL-2, IL-6, IL-7, IL-8, IL-10, IL-17, and TNFα). Positive feedback loops (especially involving IL-6) finally result in the uncontrolled release of cytokines and a cytokine storm resulting in further injury. In addition, the destruction of the gas exchange barrier leads to flooding of the alveolar space, thus increasing susceptibility to infection and finally causing lung edema and respiratory failure.

## Abbreviations

ACE2: Angiotensin-converting enzyme 2
APCs: Antigen-presenting cells
aPKC: Atypical protein kinase C
ARDS: Acute respiratory distress syndrome
AT1: Alveolar type 1
AT2: Alveolar type 2
BADJ: Bronchoalveolar-duct junctions
Ca^2+^: Calcium
CCSP: Club cell secretory protein
CLR: C-lectin type receptors
COVID-19: Coronavirus disease 2019
CoVs: Coronaviruses
CXCL1: Chemokine (C–X–C motif) ligand 1
DAG: Diacylglycerol
DAMPs: Damage-associated molecular patterns
DC-SIGN: Dendritic cell-specific intracellular adhesion molecule-3-grabbing non-integrin
ECM: Extracellular matrix
ECP: Eosinophil cationic protein
EDN: Eosinophil-derived neurotoxin
EETs: Eosinophil extracellular traps
Eph: Erythropoietin-producing hepatocellular carcinoma
Ephrins: Eph receptor interacting proteins
ERK1/2: Extracellular signal-regulated kinases 1/2
FasL: Fas ligand
FSAP: Factor VII activating protease
Gas-6: Growth arrest-specific 6
MMP-9: Matrix metalloproteinase 9 (gelatinase B)
GM-CSF: Granulocyte–macrophage colony-stimulating factor
GP Ib/IX/V: Glycoprotein Ib/IX/V
GP: Platelet glycoprotein
GPCR: G-protein-coupled seven transmembrane receptors
GRP78: Non-immune receptor glucose-regulated protein 78
HCoV-229E: Human coronavirus 229E
HCoV-HKU1: Human coronavirus HKU1
HCoV-OC43: Human coronavirus OC43
HMGB1: High-mobility group box 1 protein
ICAM-1: Intercellular adhesion molecule 1
ICTV: International Committee on Taxonomy of Viruses
IFN: Interferon
IFN-γ: Interferon gamma
Ig: Immunoglobulin
IL6: Interleukin 6
IP_3_: Inositol 1,4,5-trisphosphate
jAMs: Junctional adhesion molecules
LPS: Lipopolysaccharides
LRR receptors: Leucine-rich repeated receptors
M: Membrane glycoprotein
MAPK: Mitogen-activated protein kinase
MBP: Major basic protein
MERS-CoV: Middle East respiratory syndrome coronavirus
MGL: Macrophage galactose-type lectin
MMP2: Matrix metallopeptidase 2
MMPs: Matrix metalloproteinase
MODS: Multiple organ dysfunction syndrome
MOF: Multiorgan failure
N: Nucleocapsid
NETs: Neutrophil extracellular traps
NF-κB: Nuclear factor kappa-light-chain-enhancer of activated B cells
NK cells: Natural killer cells
NO: Nitric oxide
NOD: Nucleotide-binding oligomerization domain
NOX: Nicotinamide adenine dinucleotide phosphate oxidase
NRP1: Neuropilin-1
NSPs: Nonstructural proteins
PAF: Platelet-activating factor
PAFR: Platelet-activating factor receptor
PALS1: Protein associated with Lin-1
PAMPs: Pathogen-associated molecular patterns
PAR: Protease-activated receptor
PIP2: Phosphatidylinositol 4,5-bisphosphate
PKC: Protein kinase C
PLC2: Phospholipase C-2
pro-FSAP: Pro-factor VII activating protease
PRRs: Pattern recognition receptors
RAGE: Advanced glycation end-product
RBC: Red blood cells
ROS: Reactive oxygen species
S: Spike
SARS-CoV: Severe acute respiratory syndrome coronavirus
SARS-CoV-2: Severe acute respiratory syndrome coronavirus 2
SFK: Src family kinase
SP: Surfactant protein
TF: Tissue factor
TGF-β1: Transforming growth factor-β1
TH1: T-helper type 1
TLRs: Toll-like receptors
TMPRSS2: Transmembrane protease serine 2
TNF: Tumor necrosis factor
TNFRs: TNF receptors
TNF-α: Tumor necrosis factor-alpha
t-PA: Tissue plasminogen activator
TRAIL: TNF-related apoptosis-inducing ligand
TXA: Thromboxane
TxA2: Thromboxane A2
VAMP: Vesicle-associated membrane protein
VCAM-1: Vascular cell adhesion molecule 1
VE-cadherin: Vascular endothelial cadherin
VEGF: Vascular endothelial growth factor
VWF: Von Willebrand factor
WHO: World Health Organization
ZO: Zonula occludens

## References

[1] Zhu N, Zhang D, Wang W, Li X, Yang B, Song J (2020). China Novel Coronavirus Investigating and Research Team. A novel coronavirus from patients with pneumonia in China, 2019. N Engl J Med.

[2] Fung TS, Liu DX (2019). Human coronavirus: host–pathogen interaction. Annu Rev Microbiol.

[3] Alagheband Bahrami A, Azargoonjahromi A, Sadraei S, Aarabi A, Payandeh Z, Rajabibazl M (2022). An overview of current drugs and prophylactic vaccines for coronavirus disease 2019 (COVID-19). Cell Mol Biol Lett.

[4] Matusewicz L, Golec M, Czogalla A, Kuliczkowski K, Konka A, Zembala-John J (2022). COVID-19 therapies: do we see substantial progress?. Cell Mol Biol Lett.

[5] Letafati A, Najafi S, Mottahedi M, Karimzadeh M, Shahini A, Garousi S (2022). MicroRNA let-7 and viral infections: focus on mechanisms of action. Cell Mol Biol Lett.

[6] Saberiyan M, Karimi E, Khademi Z, Movahhed P, Safi A, Mehri-Ghahfarrokhi A (2022). SARS-CoV-2: phenotype, genotype, and characterization of different variants. Cell Mol Biol Lett.

[7] Khezri MR, Varzandeh R, Ghasemnejad-Berenji M (2022). The probable role and therapeutic potential of the PI3K/AKT signaling pathway in SARS-CoV-2 induced coagulopathy. Cell Mol Biol Lett.

[8] Kendall E, Bynoe M, Tyrrell D (1962). Virus isolations from common colds occurring in a residential school. BMJ.

[9] Ye Z-W, Yuan S, Yuen K-S, Fung S-Y, Chan C-P, Jin D-Y (2020). Zoonotic origins of human coronaviruses. Int J Biol Sci.

[10] Zhou Y, Fu B, Zheng X, Wang D, Zhao C, Qi Y (2020). Pathogenic T-cells and inflammatory monocytes incite inflammatory storms in severe COVID-19 patients. Natl Sci Rev.

[11] Drosten C, Günther S, Preiser W, Van Der Werf S, Brodt H-R, Becker S (2003). Identification of a novel coronavirus in patients with severe acute respiratory syndrome. N Engl J Med.

[12] Zaki AM, Van Boheemen S, Bestebroer TM, Osterhaus AD, Fouchier RA (2012). Isolation of a novel coronavirus from a man with pneumonia in Saudi Arabia. N Engl J Med.

[13] Singhal T (2020). A review of coronavirus disease-2019 (COVID-19). Indian J Pediatr.

[14] Wang D, Hu B, Hu C, Zhu F, Liu X, Zhang J (2020). Clinical characteristics of 138 hospitalized patients with 2019 novel coronavirus–infected pneumonia in Wuhan, China. JAMA.

[15] Edis EÇ (2020). Chronic pulmonary diseases and COVID-19. Turk Thora J.

[16] Yang X, Yu Y, Xu J, Shu H, Liu H, Wu Y (2020). Clinical course and outcomes of critically ill patients with SARS-CoV-2 pneumonia in Wuhan, China: a single-centered, retrospective, observational study. Lancet Respir Med.

[17] Mason RJ (2020). Pathogenesis of COVID-19 from a cell biology perspective. Eur Respir Soc.

[18] Choudhury A, Mukherjee S (2020). In silico studies on the comparative characterization of the interactions of SARS-CoV-2 spike glycoprotein with ACE-2 receptor homologs and human TLRs. J Med Virol.

[19] Gao C, Zeng J, Jia N, Stavenhagen K, Matsumoto Y, Zhang H (2020). SARS-CoV-2 spike protein interacts with multiple innate immune receptors. BioRxiv.

[20] Letko M, Marzi A, Munster V (2020). Functional assessment of cell entry and receptor usage for SARS-CoV-2 and other lineage B betacoronaviruses. Nat Microbiol.

[21] Matsuyama S, Nao N, Shirato K, Kawase M, Saito S, Takayama I (2020). Enhanced isolation of SARS-CoV-2 by TMPRSS2-expressing cells. Proc Natl Acad Sci USA.

[22] Tortorici MA, Walls AC, Lang Y, Wang C, Li Z, Koerhuis D (2019). Structural basis for human coronavirus attachment to sialic acid receptors. Nat Struct Mol Biol.

[23] Hilbert T, Dornbusch K, Baumgarten G, Hoeft A, Frede S, Klaschik S (2017). Pulmonary vascular inflammation: effect of TLR signalling on angiopoietin/TIE regulation. Clin Exp Pharmacol Physiol.

[24] Soker S, Takashima S, Miao HQ, Neufeld G, Klagsbrun M (1998). Neuropilin-1 is expressed by endothelial and tumor cells as an isoform-specific receptor for vascular endothelial growth factor. Cell.

[25] Ackermann M, Verleden SE, Kuehnel M, Haverich A, Welte T, Laenger F (2020). Pulmonary vascular endothelialitis, thrombosis, and angiogenesis in Covid-19. N Engl J Med.

[26] Clemetson KJ, Clemetson JM (2019). Platelet receptors.

[27] Connors JM, Levy JH (2020). Thromboinflammation and the hypercoagulability of COVID-19. J Thromb Haemost.

[28] Tang N, Li D, Wang X, Sun Z (2020). Abnormal coagulation parameters are associated with poor prognosis in patients with novel coronavirus pneumonia. J Thromb Haemost.

[29] Koupenova M, Clancy L, Corkrey HA, Freedman JE (2018). Circulating platelets as mediators of immunity, inflammation, and thrombosis. Circ Res.

[30] Bienvenu J, Monneret G, Fabien N, Revillard JP (2000). The clinical usefulness of the measurement of cytokines. Rev Res.

[31] Liu QQ, Cheng A, Wang Y, Li H, Hu L, Zhao X (2020). Cytokines and their relationship with the severity and prognosis of coronavirus disease 2019 (COVID-19): a retrospective cohort study. BMJ Open.

[32] Tang Y, Liu J, Zhang D, Xu Z, Ji J, Wen C (2020). Cytokine storm in COVID-19: the current evidence and treatment strategies. Front Immunol.

[33] Rokicki W, Rokicki M, Wojtacha J, Dżeljijli A (2016). The role and importance of club cells (Clara cells) in the pathogenesis of some respiratory diseases. Polish J Cardio Thor Surg.

[34] Stanke F (2015). The contribution of the airway epithelial cell to host defense. Mediat Inflam.

[35] Mirra V, Werner C, Santamaria F (2017). Primary ciliary dyskinesia: an update on clinical aspects, genetics, diagnosis, and future treatment strategies. Front Pediatr.

[36] Singh G, Katyal SL (1997). Clara cells and Clara cell 10 kD protein (CC10). Am J Respir Cell Mol Biol.

[37] Kia'i N, Bajaj T. 2019.Histology, respiratory epithelium. 31082105

[38] Succony L, Janes S (2014). Airway stem cells and lung cancer. Int J Med.

[39] Donne ML, Lechner AJ, Rock JR (2015). Evidence for lung epithelial stem cell niches. BMC Dev Biol.

[40] Smith A (2006). A glossary for stem-cell biology. Nature.

[41] Rock JR, Onaitis MW, Rawlins EL, Lu Y, Clark CP, Xue Y (2009). Basal cells as stem cells of the mouse trachea and human airway epithelium. Proc Natl Acad Sci USA.

[42] Giangreco A, Reynolds SD, Stripp BR (2002). Terminal bronchioles harbor a unique airway stem cell population that localizes to the bronchoalveolar duct junction. Am J Pathol.

[43] Reynolds SD, Giangreco A, Power JH, Stripp BR (2000). Neuroepithelial bodies of pulmonary airways serve as a reservoir of progenitor cells capable of epithelial regeneration. Am J Pathol.

[44] Evans M, Johnson L, Stephens R, Freeman G (1976). Renewal of the terminal bronchiolar epithelium in the rat following exposure to NO2 or O3. Lab Invest J Techn Methods Pathol.

[45] Knipe D, Howley P, Griffin D, Lamb R, Martin M, Roizman B, et al. 2013. Fields Virology, Volumes 1 and 2.

[46] Hofmann H, Geier M, Marzi A, Krumbiegel M, Peipp M, Fey GH (2004). Susceptibility to SARS coronavirus S protein-driven infection correlates with expression of angiotensin converting enzyme 2 and infection can be blocked by soluble receptor. Biochem Biophys Res Commun.

[47] Weissenhorn W, Dessen A, Calder L, Harrison S, Skehel J, Wiley D (1999). Structural basis for membrane fusion by enveloped viruses. Mol Membr Biol.

[48] Lu R, Zhao X, Li J, Niu P, Yang B, Wu H (2020). Genomic characterisation and epidemiology of 2019 novel coronavirus: implications for virus origins and receptor binding. Lancet.

[49] Jackson CB, Farzan M, Chen B, Choe H (2022). Mechanisms of SARS-CoV-2 entry into cells. Nat Rev Mol Cell Biol.

[50] Shilts J, Crozier TW, Greenwood EJ, Lehner PJ, Wright GJ (2021). No evidence for basigin/CD147 as a direct SARS-CoV-2 spike binding receptor. Sci Rep.

[51] Daly JL, Simonetti B, Klein K, Chen K-E, Williamson MK, Antón-Plágaro C (2020). Neuropilin-1 is a host factor for SARS-CoV-2 infection. Science.

[52] Cantuti-Castelvetri L, Ojha R, Pedro LD, Djannatian M, Franz J, Kuivanen S (2020). Neuropilin-1 facilitates SARS-CoV-2 cell entry and infectivity. Science.

[53] Li Z-l, Buck M (2021). Neuropilin-1 assists SARS-CoV-2 infection by stimulating the separation of Spike protein S1 and S2. Biophys J.

[54] Yan R, Zhang Y, Li Y, Xia L, Guo Y, Zhou Q (2020). Structural basis for the recognition of SARS-CoV-2 by full-length human ACE2. Science.

[55] Camargo SM, Singer D, Makrides V, Huggel K, Pos KM, Wagner CA (2009). Tissue-specific amino acid transporter partners ACE2 and collectrin differentially interact with hartnup mutations. Gastroenterology.

[56] Winstone H, Lista MJ, Reid AC, Bouton C, Pickering S, Galao RP (2021). The polybasic cleavage site in SARS-CoV-2 spike modulates viral sensitivity to type I interferon and IFITM2. J Virol.

[57] Shi G, Kenney AD, Kudryashova E, Zani A, Zhang L, Lai KK (2021). Opposing activities of IFITM proteins in SARS-CoV-2 infection. EMBO J.

[58] Zhao X, Zheng S, Chen D, Zheng M, Li X, Li G (2020). LY6E restricts entry of human coronaviruses, including currently pandemic SARS-CoV-2. J Virol.

[59] Pfaender S, Mar KB, Michailidis E, Kratzel A, Boys IN, Vkovski P (2020). LY6E impairs coronavirus fusion and confers immune control of viral disease. Nat Microbiol.

[60] Huang I-C, Bailey CC, Weyer JL, Radoshitzky SR, Becker MM, Chiang JJ (2011). Distinct patterns of IFITM-mediated restriction of filoviruses, SARS coronavirus, and influenza A virus. PLoS Pathog.

[61] Hoffmann M, Kleine-Weber H, Pöhlmann S (2020). A multibasic cleavage site in the spike protein of SARS-CoV-2 is essential for infection of human lung cells. Mol Cell.

[62] Hoffmann M, Kleine-Weber H, Schroeder S, Krüger N, Herrler T, Erichsen S (2020). SARS-CoV-2 cell entry depends on ACE2 and TMPRSS2 and is blocked by a clinically proven protease inhibitor. Cell.

[63] Masters PS (2006). The molecular biology of coronaviruses. Adv Virus Res.

[64] Fehr AR, Perlman S (2015). Coronaviruses: an overview of their replication and pathogenesis. Coronaviruses.

[65] Sawicki SG, Sawicki DL (1995). Coronaviruses use discontinuous extension for synthesis of subgenome-length negative strands. Corona Relat Virus.

[66] Chen Y, Liu Q, Guo D (2020). Emerging coronaviruses: genome structure, replication, and pathogenesis. J Med Virol.

[67] Jeffers SA, Tusell SM, Gillim-Ross L, Hemmila EM, Achenbach JE, Babcock GJ (2004). CD209L (L-SIGN) is a receptor for severe acute respiratory syndrome coronavirus. Proc Natl Acad Sci USA.

[68] Stafforini DM, McIntyre TM, Zimmerman GA, Prescott SM (2003). Platelet-activating factor, a pleiotrophic mediator of physiological and pathological processes. Crit Rev Clin Lab Sci.

[69] Marrache AM, Gobeil F, Bernier SG, Stankova J, Rola-Pleszczynski M, Choufani S (2002). Proinflammatory gene induction by platelet-activating factor mediated via its cognate nuclear receptor. J Immunol.

[70] Mukhopadhyay S, Hoidal JR, Mukherjee TK (2006). Role of TNFα in pulmonary pathophysiology. Respir Res.

[71] Bertok S, Wilson MR, Dorr AD, Dokpesi JO, O'Dea KP, Marczin N (2011). Characterization of TNF receptor subtype expression and signaling on pulmonary endothelial cells in mice. Am J Physiol Lung Cell Mol Physiol.

[72] Pokutta S, Weis WI (2007). Structure and mechanism of cadherins and catenins in cell-cell contacts. Annu Rev Cell Dev Biol.

[73] Miyahara M, Nakanishi H, Takahashi K, Satoh-Horikawa K, Tachibana K, Takai Y (2000). Interaction of nectin with afadin is necessary for its clustering at cell-cell contact sites but not for itscis dimerization or trans interaction. J Biol Chem.

[74] Lampugnani M-G, Resnati M, Raiteri M, Pigott R, Pisacane A, Houen G (1992). A novel endothelial-specific membrane protein is a marker of cell-cell contacts. J Cell Biol.

[75] Angelini DJ, Hyun S-W, Grigoryev DN, Garg P, Gong P, Singh IS (2006). TNF-α increases tyrosine phosphorylation of vascular endothelial cadherin and opens the paracellular pathway through fyn activation in human lung endothelia. Am J Physiol Lung Cell Mol Physiol.

[76] Marcos-Ramiro B, García-Weber D, Millán J (2014). TNF-induced endothelial barrier disruption: beyond actin and Rho. Thromb Haemost.

[77] Hayden MS, Ghosh S (2012). NF-κB, the first quarter-century: remarkable progress and outstanding questions. Genes Develop.

[78] Teoh K-T, Siu Y-L, Chan W-L, Schlüter MA, Liu C-J, Peiris JM (2010). The SARS coronavirus E protein interacts with PALS1 and alters tight junction formation and epithelial morphogenesis. Mol Biol Cell.

[79] Ngok SP, Geyer R, Liu M, Kourtidis A, Agrawal S, Wu C (2012). VEGF and Angiopoietin-1 exert opposing effects on cell junctions by regulating the Rho GEF Syx. J Cell Biol.

[80] De Maio F, Cascio EL, Babini G, Sali M, Della Longa S, Tilocca B, et al. 2020. Enhanced binding of SARS-CoV-2 envelope protein to tight junction-associated PALS1 could play a key role in COVID-19 pathogenesis. Review 78:346 10.1016/j.micinf.2020.08.006 PMC7473260 32891874

[81] Clark PR, Kim RK, Pober JS, Kluger MS (2015). Tumor necrosis factor disrupts claudin-5 endothelial tight junction barriers in two distinct NF-κB-dependent phases. PLoS ONE.

[82] Lee SH (2015). Intestinal permeability regulation by tight junction: implication on inflammatory bowel diseases. Intestinal research.

[83] Olson M, Kornbluth S (2001). Mitochondria in apoptosis and human disease. Curr Mol Med.

[84] Li H, Zhu H, Xu C-j, Yuan J (1998). Cleavage of BID by caspase 8 mediates the mitochondrial damage in the Fas pathway of apoptosis. Cell.

[85] Thorburn A (2004). Death receptor-induced cell killing. Cell Signal.

[86] Albertine KH, Soulier MF, Wang Z, Ishizaka A, Hashimoto S, Zimmerman GA (2002). Fas and fas ligand are up-regulated in pulmonary edema fluid and lung tissue of patients with acute lung injury and the acute respiratory distress syndrome. Am J Pathol.

[87] Wiley S (1995). Schooley K, Smolak PJ, Din WS, Huang CP, Nicholl JK, Sutherland GR, Smith TD, Rauch C, Smith CA, and Goodwin RG. Identification and characterization of a new member of the TNF family that induces apoptosis. Immunity.

[88] Rowaiye AB, Asala T, Oli AN, Uzochukwu IC, Akpa A, Esimone CO (2020). The activating receptors of natural killer cells and their inter-switching potentials. Curr Drug Targets.

[89] Soe WM, Lim JHJ, Williams DL, Goh JG, Tan Z, Sam QH (2020). Using expanded natural killer cells as therapy for invasive aspergillosis. J Fungi.

[90] Smyth MJ, Trapani JA (1995). Granzymes: exogenous proteinases that induce target cell apoptosis. Immunol Today.

[91] Griffiths GM (1995). The cell biology of CTL killing. Curr Opin Immunol.

[92] Nakajima H, Henkart PA (1994). Cytotoxic lymphocyte granzymes trigger a target cell internal disintegration pathway leading to cytolysis and DNA breakdown. J Immunol.

[93] Nagata S, Golstein P (1995). The Fas death factor. Science.

[94] Fiers W, Beyaert R, Declercq W, Vandenabeele P (1999). More than one way to die: apoptosis, necrosis and reactive oxygen damage. Oncogene.

[95] Vercammen D, Beyaert R, Denecker G, Goossens V, Van Loo G, Declercq W (1998). Inhibition of caspases increases the sensitivity of L929 cells to necrosis mediated by tumor necrosis factor. J Exp Med.

[96] Matsumura H, Shimizu Y, Ohsawa Y, Kawahara A, Uchiyama Y, Nagata S (2000). Necrotic death pathway in Fas receptor signaling. J Cell Biol.

[97] Dabrowska MI, Becks LL, Lelli JL, Levee MG, Hinshaw DB (1996). Sulfur mustard induces apoptosis and necrosis in endothelial cells. Toxicol Appl Pharmacol.

[98] Hottz ED, Bozza FA, Bozza PT (2018). Platelets in immune response to virus and immunopathology of viral infections. Front Med.

[99] Camera M, Toschi V, Brambilla M, Lettino M, Rossetti L, Canzano P (2015). The role of tissue factor in atherothrombosis and coronary artery disease: insights into platelet tissue factor. Semin Thromb Hemost.

[100] Handagama P, Scarborough RM, Shuman MA, Bainton DF (1993). Endocytosis of fibrinogen into megakaryocyte and platelet alpha-granules is mediated by alpha IIb beta 3 (glycoprotein IIb-IIIa). Blood.

[101] Thon JN, Peters CG, Machlus KR, Aslam R, Rowley J, Macleod H (2012). T granules in human platelets function in TLR9 organization and signaling. J Cell Biol.

[102] Lowenstein CJ (2017). VAMP-3 mediates platelet endocytosis. Blood.

[103] Heijnen HF, Debili N, Vainchencker W, Breton-Gorius J, Geuze HJ, Sixma JJ (1998). Multivesicular bodies are an intermediate stage in the formation of platelet α-granules. Blood J Am Soc Hematol.

[104] Koupenova M, Vitseva O, MacKay CR, Beaulieu LM, Benjamin EJ, Mick E (2014). Platelet-TLR7 mediates host survival and platelet count during viral infection in the absence of platelet-dependent thrombosis. Blood J Am Soc Hematol.

[105] Magro C, Mulvey JJ, Berlin D, Nuovo G, Salvatore S, Harp J (2020). Complement associated microvascular injury and thrombosis in the pathogenesis of severe COVID-19 infection: a report of five cases. Transl Res.

[106] Brinkmann V, Reichard U, Goosmann C, Fauler B, Uhlemann Y, Weiss DS (2004). Neutrophil extracellular traps kill bacteria. Science.

[107] Fuchs TA, Brill A, Duerschmied D, Schatzberg D, Monestier M, Myers DD (2010). Extracellular DNA traps promote thrombosis. Proc Natl Acad Sci USA.

[108] Varga Z, Flammer AJ, Steiger P, Haberecker M, Andermatt R, Zinkernagel AS (2020). Endothelial cell infection and endotheliitis in COVID-19. Lancet.

[109] Koupenova M, Kehrel BE, Corkrey HA, Freedman JE (2017). Thrombosis and platelets: an update. Eur Heart J.

[110] Golshiri K, Ataei Ataabadi E, Portilla Fernandez EC, Jan Danser A, Roks AJ (2020). The importance of the nitric oxide–cGMP pathway in age-related cardiovascular disease: focus on phosphodiesterase-1 and soluble guanylate cyclase. Basic Clin Pharmacol Toxicol.

[111] Huang C, Wang Y, Li X, Ren L, Zhao J, Hu Y (2020). Clinical features of patients infected with 2019 novel coronavirus in Wuhan. China Lancet.

[112] Bester J, Pretorius E (2016). Effects of IL-1β, IL-6 and IL-8 on erythrocytes, platelets and clot viscoelasticity. Sci Rep.

[113] Beaulieu LM, Lin E, Mick E, Koupenova M, Weinberg EO, Kramer CD (2014). Interleukin 1 receptor 1 and interleukin 1β regulate megakaryocyte maturation, platelet activation, and transcript profile during inflammation in mice and humans. Arterioscler Thromb Vasc Biol.

[114] Oleksowicz L, Mrowlec Z, Zuckerman D, Isaacs R, Dutcher J, Puszkin E (1994). Platelet activation induced by interleukin-6: evidence for a mechanism involving arachidonic acid metabolism. Thromb Haemost.

[115] AlejandroRodríguez GM, ReyesVelasco LF, Gomez J, SoleViolan J, Díaz E, Bodí M (2019). Corticosteroid treatment in critically ill patients with severe influenza pneumonia: a propensity score matching study. Score.

[116] Canzano P, Brambilla M, Porro B, Cosentino N, Tortorici E, Vicini S (2021). Platelet and endothelial activation as potential mechanisms behind the thrombotic complications of COVID-19 patients. Basic Transl Sci.

[117] Cranmer SL, Ashworth KJ, Yao Y, Berndt MC, Ruggeri ZM, Andrews RK (2011). High shear–dependent loss of membrane integrity and defective platelet adhesion following disruption of the GPIbα–filamin interaction. Blood J Am Soc Hematol.

[118] Cruz MA, Diacovo TG, Emsley J, Liddington R, Handin RI (2000). Mapping the glycoprotein Ib-binding site in the von Willebrand factor A1 domain. J Biol Chem.

[119] Du X (2007). Signaling and regulation of the platelet glycoprotein Ib–IX–V complex. Curr Opin Hematol.

[120] Li Z, Zhang G, Feil R, Han J, Du X (2006). Sequential activation of p38 and ERK pathways by cGMP-dependent protein kinase leading to activation of the platelet integrin αIIbβ3. Blood.

[121] Li Z, Xi X, Gu M, Feil R, Richard DY, Eigenthaler M (2003). A stimulatory role for cGMP-dependent protein kinase in platelet activation. Cell.

[122] Garcia A, Quinton TM, Dorsam RT, Kunapuli SP (2005). Src family kinase–mediated and Erk-mediated thromboxane A2 generation are essential for VWF/GPIb-induced fibrinogen receptor activation in human platelets. Blood.

[123] Gibbins JM, Okuma M, Farndale R, Barnes M, Watson SP (1997). Glycoprotein VI is the collagen receptor in platelets which underlies tyrosine phosphorylation of the Fc receptor γ-chain. FEBS Lett.

[124] Ezumi Y, Shindoh K, Tsuji M, Takayama H (1998). Physical and functional association of the Src family kinases Fyn and Lyn with the collagen receptor glycoprotein VI-Fc receptor γ chain complex on human platelets. J Exp Med.

[125] Harper M, Poole A (2010). Diverse functions of protein kinase C isoforms in platelet activation and thrombus formation. J Thromb Haemost.

[126] Varga-Szabo D, Braun A, Nieswandt B (2009). Calcium signaling in platelets. J Thromb Haemost.

[127] Blair P, Rex S, Vitseva O, Beaulieu L, Tanriverdi K, Chakrabarti S (2009). Stimulation of Toll-like receptor 2 in human platelets induces a thromboinflammatory response through activation of phosphoinositide 3-kinase. Circ Res.

[128] Panigrahi S, Ma Y, Hong L, Gao D, West XZ, Salomon RG (2013). Engagement of platelet toll-like receptor 9 by novel endogenous ligands promotes platelet hyperreactivity and thrombosis. Circ Res.

[129] Akira S, Uematsu S, Takeuchi O (2006). Pathogen recognition and innate immunity. Cell.

[130] Zhang G, Han J, Welch EJ, Richard DY, Voyno-Yasenetskaya TA, Malik AB (2009). Lipopolysaccharide stimulates platelet secretion and potentiates platelet aggregation via TLR4/MyD88 and the cGMP-dependent protein kinase pathway. J Immunol.

[131] Müller S, Ronfani L, Bianchi M (2004). Regulated expression and subcellular localization of HMGB1, a chromatin protein with a cytokine function. J Intern Med.

[132] Coughlin SR (2005). Protease-activated receptors in hemostasis, thrombosis and vascular biology. J Thromb Haemost.

[133] De Candia E, Hall SW, Rutella S, Landolfi R, Andrews RK, De Cristofaro R (2001). Binding of thrombin to glycoprotein Ib accelerates the hydrolysis of Par-1 on intact platelets. J Biol Chem.

[134] Estevez B, Kim K, Delaney MK, Stojanovic-Terpo A, Shen B, Ruan C (2016). Signaling-mediated cooperativity between glycoprotein Ib-IX and protease-activated receptors in thrombin-induced platelet activation. Blood J Am Soc Hematol.

[135] Yin H, Liu J, Li Z, Berndt MC, Lowell CA, Du X (2008). Src family tyrosine kinase Lyn mediates VWF/GPIb-IX–induced platelet activation via the cGMP signaling pathway. Blood.

[136] Delaney MK, Kim K, Estevez B, Xu Z, Stojanovic-Terpo A, Shen B (2016). Differential roles of the NADPH-oxidase 1 and 2 in platelet activation and thrombosis. Arterioscler Thromb Vasc Biol.

[137] Watanabe N, Nakajima H, Suzuki H, Oda A, Matsubara Y, Moroi M (2003). Functional phenotype of phosphoinositide 3-kinase p85α-null platelets characterized by an impaired response to GP VI stimulation. Blood.

[138] Lian L, Wang Y, Draznin J, Eslin D, Bennett JS, Poncz M (2005). The relative role of PLCβ and PI3Kγ in platelet activation. Blood.

[139] Minuz P, Meneguzzi A, Fumagalli L, Degan M, Calabria S, Ferraro R (2018). Calcium-dependent src phosphorylation and reactive oxygen species generation are implicated in the activation of human platelet induced by thromboxane a2 analogs. Front Pharmacol.

[140] Estevez B, Shen B, Du X (2015). Targeting integrin and integrin signaling in treating thrombosis. Arterioscler Thromb Vasc Biol.

[141] Li Z, Delaney MK, O'Brien KA, Du X (2010). Signaling during platelet adhesion and activation. Arterioscler Thromb Vasc Biol.

[142] Brosnahan SB, Jonkman AH, Kugler MC, Munger JS, Kaufman DA (2020). COVID-19 and respiratory system disorders: current knowledge, future clinical and translational research questions. Arterioscler Thromb Vasc Biol.

[143] Urano T, Suzuki Y (2012). Accelerated fibrinolysis and its propagation on vascular endothelial cells by secreted and retained tPA. J Biomed Biotechnol.

[144] Zuo Y, Warnock M, Harbaugh A, Yalavarthi S, Gockman K, Zuo M (2021). Plasma tissue plasminogen activator and plasminogen activator inhibitor-1 in hospitalized COVID-19 patients. Sci Rep.

[145] Ning M, Furie K, Koroshetz W, Lee H, Barron M, Lederer M (2006). Association between tPA therapy and raised early matrix metalloproteinase-9 in acute stroke. Neurology.

[146] Rauch I, Iglseder B, Paulweber B, Ladurner G, Strasser P (2008). MMP-9 haplotypes and carotid artery atherosclerosis: an association study introducing a novel multicolour multiplex RealTime PCR protocol. Eur J Clin Investig.

[147] Parsons S, Watson S, Brown P, Collins H, Steele R (1997). Matrix metalloproteinases. J Br Surg.

[148] Lin T-C, Li C-Y, Tsai C-S, Ku C-H, Wu C-T, Wong C-S (2005). Neutrophil-mediated secretion and activation of matrix metalloproteinase-9 during cardiac surgery with cardiopulmonary bypass. Anesth Analg.

[149] Jesenak M, Brndiarova M, Urbancikova I, Rennerova Z, Vojtkova J, Bobcakova A (2020). Immune parameters and COVID-19 infection—associations with clinical severity and diseases prognosis. Front Cell Infect Microbiol.

[150] Yao X, Li T, He Z, Ping Y, Liu H, Yu S (2020). A pathological report of three COVID-19 cases by minimally invasive autopsies. Chin J Pathol.

[151] Narasaraju T, Yang E, Samy RP, Ng HH, Poh WP, Liew A-A (2011). Excessive neutrophils and neutrophil extracellular traps contribute to acute lung injury of influenza pneumonitis. Am J Pathol.

[152] Yamamichi S, Fujiwara Y, Kikuchi T, Nishitani M, Matsushita Y, Hasumi K (2011). Extracellular histone induces plasma hyaluronan-binding protein (factor VII activating protease) activation in vivo. Biochem Biophys Res Commun.

[153] Wolberg AS, Aleman MM (2010). Influence of cellular and plasma procoagulant activity on the fibrin network. Thromb Res.

[154] Fuchs TA, Bhandari AA, Wagner DD (2011). Histones induce rapid and profound thrombocytopenia in mice. Blood.

[155] Massberg S, Grahl L, von Bruehl M-L, Manukyan D, Pfeiler S, Goosmann C (2010). Reciprocal coupling of coagulation and innate immunity via neutrophil serine proteases. Nat Med.

[156] Ward SE, Curley GF, Lavin M, Fogarty H, Karampini E, McEvoy NL (2021). Von Willebrand factor propeptide in severe coronavirus disease 2019 (COVID-19): evidence of acute and sustained endothelial cell activation. Br J Haematol.

[157] Tomokiyo K, Kamikubo Y, Hanada T, Araki T, Nakatomi Y, Ogata Y (2005). Von Willebrand factor accelerates platelet adhesion and thrombus formation on a collagen surface in platelet-reduced blood under flow conditions. Blood.

[158] Denzel A, Maus UA, Gomez MR, Moll C, Niedermeier M, Winter C (2008). Basophils enhance immunological memory responses. Nat Immunol.

[159] Redrup AC, Howard BP, MacGlashan DW, Kagey-Sobotka A, Lichtenstein LM, Schroeder JT (1998). Differential regulation of IL-4 and IL-13 secretion by human basophils: their relationship to histamine release in mixed leukocyte cultures. J Immunol.

[160] Lai EC (2004). Notch signaling: control of cell communication and cell fate. Development.

[161] Yabe Y, Matsumoto T, Tsurumoto T, Shindo H (2005). Immunohistological localization of Notch receptors and their ligands Delta and Jagged in synovial tissues of rheumatoid arthritis. J Orthop Sci.

[162] Aster JC, Pear WS, Blacklow SC (2017). The varied roles of notch in cancer. Annu Rev Pathol.

[163] Gratton R, Tricarico PM, d'Adamo AP, Bianco AM, Moura R, Agrelli A (2020). Notch signaling regulation in autoinflammatory diseases. Int J Mol Sci.

[164] Webb LM, Oyesola OO, Früh SP, Kamynina E, Still KM, Patel RK (2019). The Notch signaling pathway promotes basophil responses during helminth-induced type 2 inflammation. J Exp Med.

[165] Osborne BA, Minter LM (2007). Notch signalling during peripheral T-cell activation and differentiation. Nat Rev Immunol.

[166] Hoyne GF, Le Roux I, Corsin-Jimenez M, Tan K, Dunne J, Forsyth LM (2000). Serrate1-induced Notch signalling regulates the decision between immunity and tolerance made by peripheral CD4+ T cells. Int Immunol.

[167] Popi AF, Longo-Maugéri IM, Mariano M (2016). An overview of B-1 cells as antigen-presenting cells. Front Immunol.

[168] Drake MG, Bivins-Smith ER, Proskocil BJ, Nie Z, Scott GD, Lee JJ (2016). Human and mouse eosinophils have antiviral activity against parainfluenza virus. Am J Respir Cell Mol Biol.

[169] Handzel ZT, Busse WW, Sedgwick JB, Vrtis R, Lee WM, Kelly E (1998). Eosinophils bind rhinovirus and activate virus-specific T cells. J Immunol.

[170] Bredt D, Snyder SH (1994). Nitric oxide: a physiologic messenger molecule. Annu Rev Biochem.

[171] Renner A, Marth K, Patocka K, Idzko M, Pohl W (2020). COVID-19 in two severe asthmatics receiving benralizumab: busting the eosinophilia myth. ERJ Open Res.

[172] Yousefi S, Simon D, Simon H-U (2012). Eosinophil extracellular DNA traps: molecular mechanisms and potential roles in disease. Curr Opin Immunol.

[173] Temizoz B, Kuroda E, Ohata K, Jounai N, Ozasa K, Kobiyama K (2015). TLR9 and STING agonists synergistically induce innate and adaptive type-II IFN. Eur J Immunol.

[174] Dreyfus G (1999). University becomes political football. Nature.

[175] Spencer LA, Szela CT, Perez SA, Kirchhoffer CL, Neves JS, Radke AL (2009). Human eosinophils constitutively express multiple Th1, Th2, and immunoregulatory cytokines that are secreted rapidly and differentially. J Leukoc Biol.

[176] Wassom D, Loegering D, Solley G, Moore S, Schooley R, Fauci A (1981). Elevated serum levels of the eosinophil granule major basic protein in patients with eosinophilia. J Clin Investig.

[177] Pflanz S, Hibbert L, Mattson J, Rosales R, Vaisberg E, Bazan JF (2004). WSX-1 and glycoprotein 130 constitute a signal-transducing receptor for IL-27. J Immunol.

[178] Kaplanski G (2018). Interleukin-18: biological properties and role in disease pathogenesis. Immunol Rev.

[179] Presky DH, Yang H, Minetti LJ, Chua AO, Nabavi N, Wu C-Y (1996). A functional interleukin 12 receptor complex is composed of two β-type cytokine receptor subunits. Proc Natl Acad Sci USA.

[180] Choi YH, Lim EJ, Kim SW, Moon YW, Park KS, An H-J (2019). IL-27 enhances IL-15/IL-18-mediated activation of human natural killer cells. J Immunother Cancer.

[181] Dodge IL, Carr MW, Cernadas M, Brenner MB (2003). IL-6 production by pulmonary dendritic cells impedes Th1 immune responses. J Immunol.

[182] Navarro S, Aleu J, Jimenez M, Boix E, Cuchillo C, Nogues M (2008). The cytotoxicity of eosinophil cationic protein/ribonuclease 3 on eukaryotic cell lines takes place through its aggregation on the cell membrane. Cell Mol Life Sci.

[183] Theoharides TC. COVID‐19, pulmonary mast cells, cytokine storms, and beneficial actions of luteolin. Biofactors (Oxford, England). 2020. 10.1002/biof.1633 PMC7267424 32339387

[184] Lanier LL (2008). Up on the tightrope: natural killer cell activation and inhibition. Nat Immunol.

[185] Saddawi-Konefka R, Seelige R, Gross ET, Levy E, Searles SC, Washington A (2016). Nrf2 induces IL-17D to mediate tumor and virus surveillance. Cell Rep.

[186] Sannohe S, Adachi T, Hamada K, Honda K, Yamada Y, Saito N (2003). Upregulated response to chemokines in oxidative metabolism of eosinophils in asthma and allergic rhinitis. Eur Respir J.

[187] Bartemes KR, McKinney S, Gleich GJ, Kita H (1999). Endogenous platelet-activating factor is critically involved in effector functions of eosinophils stimulated with IL-5 or IgG. J Immunol.

[188] Kellner M, Noonepalle S, Lu Q, Srivastava A, Zemskov E, Black SM. ROS signaling in the pathogenesis of acute lung injury (ALI) and acute respiratory distress syndrome (ARDS). Pulmonary Vasculature Redox Signaling in Health and Disease: Springer; 2017. p. 105–37. 10.1007/978-3-319-63245-2_8 PMC7120947 29047084

[189] Cifaldi L, Prencipe G, Caiello I, Bracaglia C, Locatelli F, De Benedetti F (2015). Inhibition of natural killer cell cytotoxicity by interleukin-6: implications for the pathogenesis of macrophage activation syndrome. Arthrit Rheumatol.

[190] Hunter CA, Timans J, Pisacane P, Menon S, Cai G, Walker W (1997). Comparison of the effects of interleukin-1α, interleukin-lβ and interferon-γ-inducing factor on the production of interferon-γ by natural killer. Eur J Immunol.

[191] Zhou Y, Fu B, Zheng X, Wang D (2020). Zhao Ch Qi Y Pathogenic T cells and inflammatory monocytes incite inflammatory storm in severe COVID-19 patients. Natl Sci Rev.

[192] Bradley M, Zeytun A, Rafi-Janajreh A, Nagarkatti PS, Nagarkatti M (1998). Role of spontaneous and interleukin-2–induced natural killer cell activity in the cytotoxicity and rejection of Fas^+^ and Fas^−^ tumor cells. Blood J Am Soc Hematol.

[193] Screpanti V, Wallin RP, Ljunggren H-G, Grandien A (2001). A central role for death receptor-mediated apoptosis in the rejection of tumors by NK cells. J Immunol.

[194] Goodnow CC, Vinuesa CG, Randall KL, Mackay F, Brink R (2010). Control systems and decision making for antibody production. Nat Immunol.

[195] Schultze JL, Michalak S, Lowne J, Wong A, Gilleece MH, Gribben JG (1999). Human non-germinal center B cell interleukin (IL)-12 production is primarily regulated by T cell signals CD40 ligand, interferon γ, and IL-10: role of B cells in the maintenance of T cell responses. J Exp Med.

[196] Dewald JH, Colomb F, Bobowski-Gerard M, Groux-Degroote S, Delannoy P (2016). Role of cytokine-induced glycosylation changes in regulating cell interactions and cell signaling in inflammatory diseases and cancer. Cells.

[197] Arbour N, Beauseigle D, Duquette P, Prat A, Schneider R, Mohebiany AN, et al. 2011. B cell-derived IL-15 enhances CD8 T cell. 10.4049/jimmunol.1100885 PMC5052068 21911607

[198] Abbasifard M, Kamiab Z, Hasani M, Rahnama A, Saeed-Askari P, Khorramdelazad H (2020). Assessing the expression of immunosuppressive cytokines in the newly diagnosed systemic lupus erythematosus patients: a focus on B cells. BMC Immunol.

[199] Munn DH, Zhou M, Attwood JT, Bondarev I, Conway SJ, Marshall B (1998). Prevention of allogeneic fetal rejection by tryptophan catabolism. Science.

[200] Li R, Rezk A, Miyazaki Y, Hilgenberg E, Touil H, Shen P (2015). Science translational medicine. Sci Transl Med.

[201] Nimer SD, Uchida H (1995). Regulation of granulocyte–macrophage colony-stimulating factor and interleukin 3 expression. Stem Cells.

[202] Shibata Y, Berclaz P-Y, Chroneos ZC, Yoshida M, Whitsett JA, Trapnell BC (2001). GM-CSF regulates alveolar macrophage differentiation and innate immunity in the lung through PU. Immunity.

[203] Nahrendorf M, Swirski FK (2013). Monocyte and macrophage heterogeneity in the heart. Circ Res.

[204] Buzon MJ, Seiss K, Weiss R, Brass AL, Rosenberg ES, Pereyra F (2011). Inhibition of HIV-1 integration in ex vivo-infected CD4 T cells from elite controllers. J Virol.

[205] Fadok VA, Bratton DL, Konowal A, Freed PW, Westcott JY, Henson PM (1998). Macrophages that have ingested apoptotic cells in vitro inhibit proinflammatory cytokine production through autocrine/paracrine mechanisms involving TGF-beta, PGE2, and PAF. J Clin Investig.

[206] Liu D, Du L, Chen D, Ye Z, Duan H, Tu T (2016). Reduced CD146 expression promotes tumorigenesis and cancer stemness in colorectal cancer through activating Wnt/β-catenin signaling. Oncotarget.

[207] Malik B, Abdelazeem B, Ghatol A (2021). Pulmonary fibrosis after COVID-19 pneumonia. Cureus.

[208] Romagnani S (2000). T-cell subsets (Th1 versus Th2). Ann Allergy Asthma Immunol.

[209] Mattiola I, Pesant M, Tentorio PF, Molgora M, Marcenaro E, Lugli E (2015). Priming of human resting NK cells by autologous M1 macrophages via the engagement of IL-1β, IFN-β, and IL-15 pathways. J Immunol.

[210] Garcia J, Rodriguez F, De Cabo M, Salgado M, Losada J, Villaron L (1999). Evaluation of inflammatory cytokine secretion by human alveolar macrophages. Mediat Inflamm.

[211] Crouch E, Wright JR (2001). Surfactant proteins A and D and pulmonary host defense. Annu Rev Physiol.

[212] Fehrenbach H (2001). Alveolar epithelial type II cell: defender of the alveolus revisited. Respir Res.

